# Selective inhibition of STAT3 signaling using monobodies targeting the coiled-coil and N-terminal domains

**DOI:** 10.1038/s41467-020-17920-z

**Published:** 2020-08-17

**Authors:** Grégory La Sala, Camille Michiels, Tim Kükenshöner, Tania Brandstoetter, Barbara Maurer, Akiko Koide, Kelvin Lau, Florence Pojer, Shohei Koide, Veronika Sexl, Laure Dumoutier, Oliver Hantschel

**Affiliations:** 1 Swiss Institute for Experimental Cancer Research (ISREC), School of Life Sciences, École polytechnique fédérale de Lausanne (EPFL), Station 19, 1015 Lausanne, Switzerland; 2 Experimental Medicine Unit, De Duve Institute, Université catholique de Louvain, 1200 Brussels, Belgium; 3 Institute of Pharmacology and Toxicology, University of Veterinary Medicine, Vienna, Austria; 4 Department of Medicine, New York University School of Medicine, 522 1st Avenue, New York, 10016 NY USA; 5 Laura and Isaac Perlmutter Cancer Center, New York University Langone Health, 522 1st Avenue, New York, 10016 NY USA; 6 Protein Crystallography Core Facility, School of Life Sciences, École polytechnique fédérale de Lausanne, Station 19, 1015 Lausanne, Switzerland; 7 Department of Biochemistry and Molecular Pharmacology, New York University School of Medicine, 522 1st Avenue, New York, 10016 NY USA; 8 Faculty of Medicine, Institute of Physiological Chemistry, Philipps-University of Marburg, Karl-von-Frisch-Straße 1, 35032 Marburg, Germany

**Keywords:** Oncogene proteins, Transcription factors, Antibody fragment therapy, X-ray crystallography

## Abstract

The transcription factor STAT3 is frequently activated in human solid and hematological malignancies and remains a challenging therapeutic target with no approved drugs to date. Here, we develop synthetic antibody mimetics, termed monobodies, to interfere with STAT3 signaling. These monobodies are highly selective for STAT3 and bind with nanomolar affinity to the N-terminal and coiled-coil domains. Interactome analysis detects no significant binding to other STATs or additional off-target proteins, confirming their exquisite specificity. Intracellular expression of monobodies fused to VHL, an E3 ubiquitin ligase substrate receptor, results in degradation of endogenous STAT3. The crystal structure of STAT3 in complex with monobody MS3-6 reveals bending of the coiled-coil domain, resulting in diminished DNA binding and nuclear translocation. MS3-6 expression strongly inhibits STAT3-dependent transcriptional activation and disrupts STAT3 interaction with the IL-22 receptor. Therefore, our study establishes innovative tools to interfere with STAT3 signaling by different molecular mechanisms.

## Abstract (web summary)

STAT3 is an attractive therapeutic target but its homology with other STAT proteins complicates the development of selective inhibitors. Here, the authors develop monobodies with high affinity and selectivity for STAT3 and show that they can interfere with cellular STAT3 activity.

## Introduction

The signal transducer and activator of transcription (STAT) family is composed of STAT1-4, STAT5A, STAT5B, and STAT6 and is implicated in inflammation, proliferation, differentiation, cell survival and immune responses^1^. These responses are driven by cytokines stimulating receptors upstream of the Janus kinases (JAKs) that are responsible for the phosphorylation of STAT proteins^2,3^. This signal leads to the dimerization of STATs mediated by their SH2 domains and phospho-tyrosine residues. STAT dimers then translocate into the nucleus and bind specific DNA sequences to regulate target gene expression^4,5^.

STAT3 is ubiquitously expressed and activated by various interleukins, interferons, growth factors, as well as directly by oncogenic kinases such as Src, Abl or Met^6–9^. Additionally, STAT3 is frequently activated in human cancers^10–13^ and is implicated in the regulation of cancer cell survival, proliferation, angiogenesis and metastasis^4,14,15^. Gain-of-function mutations of STAT3 have recently been identified in patients suffering from various haematological malignancies and found to be associated with reduced overall survival^16,17^. Hence, the direct therapeutic inhibition of STAT3 is highly desirable but remains challenging as evident from the lack of FDA approved drugs^18,19^.

**Fig. 1:**
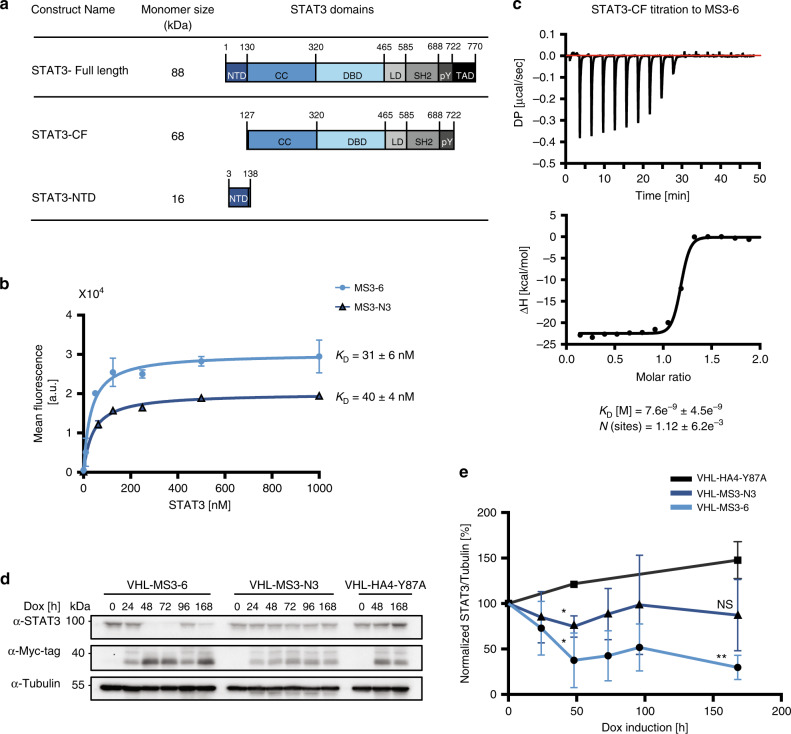
Selection of high affinity, STAT3-selective monobody binders. **a** Schematic representation of the recombinant STAT3 constructs used for monobody selection. **b** Binding titration of yeast cells displaying monobodies (MS3-6: light blue line, MS3-N3: dark blue line) at their surface to recombinant STAT3 proteins. The mean fluorescence intensity of yeast cells bound to the target are plotted as a function of the protein concentration. Data from three individual experiments, mean ± SD are shown and a curve fitting 1:1 binding model was used. **c** Isothermal calorimetric titration (ITC) of STAT3 (100 µM) to a MS3-6 solution (10 µM) performed at 25 °C. The upper panel shows raw heat signal, while the lower panel shows the integrated calorimetric data of the area for each peak. A best fit 1:1 binding model was used and is illustrated by a black line (Microcal software). *K*_D_ and stoichiometry values (N) are indicated in the figure. ∆*H* (kcal mol^−1^) = −22.5 ± 0.5; ∆G (kcal mol^−1^) = −11.1. **d** Representative immunoblot analysis from three independent experiments of monobody-VHL fusion expression overtime upon doxycycline treatment (1 µg ml^−1^) leading to STAT3 degradation. **e** STAT3 degradation levels were quantified and plotted from three independent experiments. Mean ± SD are shown and significance is indicated according to a two-sided unpaired *t-*test against HA4-Y87A control: MS3-6 *P = 0.033, **P = 0.0011, MS3-N3 *P = 0.0125. Source data are provided as a source data file.

STAT3 shares sequence and structural homology with other STAT family members and is composed of six domains: N-terminal domain (NTD), coiled-coil domain (CC), DNA-binding domain (DBD), linker domain (LD), Src homology 2 domain (SH2) and transactivation domain (TAD, Fig. 1a)^20^. Canonical STAT3 activation relies on the phosphorylation of a key tyrosine residue by JAKs, Y705, which results in SH2 domain-mediated STAT3 dimerization^21^. Most inhibitory approaches aim to preclude STAT3 dimerization, but they have shown only limited clinical efficacy so far^3^. By contrast, the inhibition of STAT3 by targeting other domains remains largely unexplored, although the CC and NTD are also implicated in mediating critical functions of STAT3. The NTD mediates the recognition of weaker DNA binding sites and preferentially mediates the formation of an anti-parallel dimer of un-phosphorylated STAT3^20,22,23^, whereas the CC is important for nuclear translocation^24–26^. Additionally, the CC domain of STAT3 has a pivotal role in IL-22 receptor (IL-22R) signaling, where it constitutively associates with the C-terminal region of IL-22R and leads to a non-conventional activation of STAT3 that is independent of phospho-tyrosines on the IL-22R^27^. This mechanism complements the conventional phospho-tyrosine residue-dependent IL-22R/STAT3 activation mechanism^27^. IL-22 signaling is a potent mediator of cellular inflammatory responses and is unique among the cytokines, as it only acts on non-hematopoietic stromal cells^28^. The IL-22 signaling axis is a key player in chronic inflammatory disorders, such as psoriasis, but also promotes tumor growth, metastasis and inhibition of apoptosis^28^. Therefore, achieving a specific blockade of alternative STAT3 activation in IL-22R signaling is of particular interest.

Monobodies are synthetic binding proteins, which are built using the tenth fibronectin type III (FN3) domain of human fibronectin as the molecular scaffold^29^. They are obtained from large combinatorial libraries by sequential phage and yeast display selection^30,31^. Monobodies can be selected to bind to proteins that lack deep catalytic grooves, which are difficult to target by small molecules^32^. Over the past decade, monobodies targeting different oncoproteins, such as BCR-ABL^33,34^, SRC^35^ and Aurora A kinases^36^, the SHP2 phosphatase^37^ and the RAS GTPase^38^ have been developed. Intracellular expression of these monobodies resulted in inhibition of oncogenic signaling and cell proliferation. These studies established monobodies as highly specific intracellular antagonists and their utility in advancing a mechanistic understanding of targeted proteins and in discovering new sites for interfering with their functions^39,40^. Nonetheless, no monobodies antagonizing a transcription factor, which were deemed as untargetable proteins for decades, are yet available.

Here, we develop monobodies to different STAT3 domains to interfere with critical cellular and pathophysiological processes. The monobodies bind the N-terminal or coiled-coil domain with high affinity and selectivity, and inhibit the transcriptional activity of STAT3. We present a crystal structure of a lead monobody MS3-6 bound to the CC domain of STAT3 and insights on its molecular mode of action in interfering with conventional and non-conventional STAT3 activation. Our results demonstrate the utility of blocking intracellular STAT3 signaling upon targeting of the therapeutically unexplored CC domain of STAT3 and pave a way towards inhibitory strategies in diverse diseases including cancer and autoimmune disorders.

## Results

### Generation of high affinity monobodies targeting STAT3

To generate monobody binders against human STAT3, we first recombinantly expressed and purified the core fragment (CF) of STAT3, which encompasses the CC, DBD and SH2 domains (STAT3-CF, a.a. 127–722, Fig. 1a), as well as the N-terminal domain (NTD, a.a. 3–138; Fig. 1a). Both recombinant proteins were confirmed to be of high purity and monomeric in solution (Supplementary Fig. 1a, b). We identified monobodies from the combinatorial side-and-loop library using phage and yeast display library sorting^30,31,33^. Seven monobody clones binding to STAT3-CF and five clones binding to STAT3-NTD were isolated and further characterized (Table 1). The recombinant monobodies expressed in *E. coli* with high yield and solubility and size exclusion chromatography coupled to multi-angle light scattering (SEC-MALS) indicated the formation of a monodisperse 1:1 complex with STAT3-CF when the two components were mixed (Supplementary Fig. 1c, d). In order to measure the binding affinities to their respective targets, increasing concentrations of recombinant STAT3 proteins were titrated to yeast cells displaying the monobodies. Each clone exhibited an apparent dissociation constant (*K*_D_) in the mid-nanomolar range (Table 1 and Supplementary Fig. 1e). Monobody MS3-6 had the highest apparent affinity toward STAT3-CF (31 ± 6 nM). For the STAT3-NTD, several clones showed comparably high affinities. Of those, monobody MS3-N3 had the highest solubility with an apparent binding affinity of 40 ± 4 nM (Table 1 and Fig. 1b). Interestingly, competition assays for STAT3 binding in the presence of MS3-6 showed that all STAT3-CF monobodies share an overlapping or possibly common epitope despite their different sequences (Supplementary Fig. 1f). We thus concentrated our efforts on the characterization of the MS3-6 monobody. The thermodynamics of MS3-6 binding to STAT3-CF was investigated using isothermal titration calorimetry (ITC). We found a dominantly enthalpically driven binding (∆*H* = −22.5 ± 0.5 kcal/mol) with a binding stoichiometry of 1:1 and an ensuing high binding affinity (*K*_*D*_ = 7.6 ± 4.5 nM; Fig. 1c). Over the past decade, a number of STAT3 oncogenic mutants have been identified in patients suffering from solid and hematological malignancies^41^. MS3-6 bound recombinant STAT3-CF proteins harboring the S614R and D661V oncogenic mutations in their SH2 domain, as well as the Y705F dominant negative mutant, with apparent *K*_*D*_ values of 94.7 ± 16 nM, 18.5 ± 6 and 17.6 ± 7 nM, respectively, in yeast display binding assays, which was in a comparable range to the wildtype STAT3 protein (Supplementary Fig. 1g).

**Table 1:**
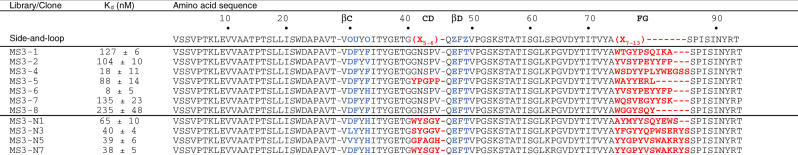
Original monobody library and selected STAT3 binders.

Amino acid sequences of the side-and-loop monobody library are shown on top, where “X” corresponds to a blend of 30% Tyr, 15% Ser, 10% Gly, 5% Phe, 5% Trp and 2.5% each of all remaining amino acids with the exception of Cys. “B” denotes a mixture of Gly, Ser and Tyr. “J” corresponds to a mixture of Ser and Tyr; “O”, a mixture of Asn, Asp, His, Ile, Leu, Phe, Tyr and Val. “U”, a mixture of His, Phe and Tyr. “Z”, Ala, Glu, Lys and Thr. Monobodies selected against STAT3-CF are indicated in the upper panel (MS3-1 to MS3-8), while monobodies selected against STAT3-NTD are shown in the lower panel (MS3-N1 to MS3-N7). Dissociation constants (*K*_D_) are indicated according to yeast binding assays. Binding curves are shown in Supplementary Fig. 1e.

In summary, we have developed monobodies that bind with high affinity either to the STAT3-CF or the STAT3-NTD, and that are capable of binding to oncogenic STAT3 mutants.

### STAT3 monobodies are selective over other STATs in cells

Due to the high sequence and structural conservation among the STAT family members, the development of selective chemical probes remains challenging. To address selectivity of the developed STAT3 monobodies, we assayed binding of MS3-6 to the recombinant core fragment of STAT5B (a.a. 129–714), which encouragingly could not be detected in vitro (Supplementary Fig. 1g). To evaluate whether MS3-6 and MS3-N3 are able to bind to STAT3 in the complex cellular environment of mammalian cells, we transiently transfected HEK293 cells with 6xMyc-tagged monobodies and tested for binding to endogenous STAT3. Enrichment of STAT3 in the Myc-tag pull down fraction was observed by immunoblot suggesting an effective binding between STAT3 and MS3-6 or MS3-N3 in cells (Supplementary Fig. 2a).

To assess functional engagement of STAT3 by the selected monobodies in cells, we engineered a targeted degradation system by fusing the monobodies to the Von Hippel−Lindau (VHL) protein^42,43^, which is the substrate receptor of the Cullin2/RBX1 E3 ubiquitin ligase complex. Inducible expression of MS3-6-VHL fusions in NPM-ALK expressing mouse thymoma cells resulted in the degradation of endogenous STAT3 protein (Fig. 1d, e). To a lower extend, MS3-N3-VHL expression led to the degradation of STAT3 over time as compared to the non-binding HA4-Y87A-VHL control (Fig. 1d, e). STAT3 degradation by MS3-6-VHL was impaired upon proteasome inhibition, indicating ubiquitin-dependent proteasomal degradation (Supplementary Fig. 2b). These results show that the monobodies mediated a targeted degradation of STAT3 in cells, thus highlighting successful target engagement.

To comprehensively assess the selectivity of MS3-6 and MS3-N3 in an unbiased way on a proteome-wide scale, we performed monobody affinity-purification mass spectrometry experiments^37,44^. Tandem affinity purification (TAP)-tagged monobodies were stably expressed in two cell lines that endogenously express STAT3 and various other STAT family members at different levels: K562 chronic myeloid leukemia cells and Jurkat acute lymphoblastic leukemia T-cells. Immunoblot analysis of fractions from the TAPs of ~10^9^ cells each showed that both the monobody and its on-target STAT3 were efficiently recovered after the second affinity purification step (Supplementary Fig. 2c). The composition of the recovered fractions was assessed by SDS-PAGE analysis (Supplementary Fig. 2d) and identified two main bands at ~20 kDa and ~80 kDa corresponding to the molecular weight of the monobodies and STAT3, respectively. Subsequent liquid chromatography coupled to tandem mass spectrometry (LC–MS/MS) analysis identified both the monobody itself and full-length STAT3 to be highly abundant among the identified proteins (Table 2). In contrast, only very low levels of STAT1 and STAT5B were identified in both cell lines. Other STATs were not detected despite expression in both K562 and Jurkat cells (Table 2 and Supplementary Fig. 2e). For MS3-N3, abundance of STAT3 was overall lower, but either no other STATs were detected (K562 cells) or much less abundant (STAT5B in Jurkat; Table 2). The modest abundance of proteins other than STAT3 isoforms, their inconsistent identification across the purifications and general high cellular abundance suggest that these are nonspecifically recovered in the TAP experiments, rather than being true off-targets of these monobodies.

**Table 2:** Monobody MS3-6 and MS3-N3 interactome.

Bait monobody	Cell line	STAT family proteins identified^a^	Spectrum counts of razor and unique peptides^b^	iBAQ (intensities)^c^	Rank out of all identified proteins^d^
MS3-6					
	Jurkat				
		Monobody	498	6.74 × 10^9^	2/578
		STAT3-FL	599	1.23 × 10^9^	6/578
		STAT3 Del-701	7	1.22 × 10^9^	7/578
		STAT3-β	3	1.21 × 10^9^	8/578
		STAT1	2	4.52 × 10^5^	425/578
	K562				
		Monobody	1967	1.38 × 10^10^	1/566
		STAT3 [a.a. 3–84]	5	5.05 × 10^9^	3/566
		STAT3-FL	3318	3.11 × 10^9^	4/566
		STAT3-β	14	3.11 × 10^9^	5/566
		STAT3 Del-701	29	2.99 × 10^9^	6/566
		STAT1	7	2.56 × 10^5^	253/566
		STAT5B	5	1.55 × 10^5^	321/566
MS3-N3					
	Jurkat				
		Monobody	62	8,43 × 10^9^	3/399
		STAT3-FL	173	4.63 × 10^7^	41/399
		STAT3 Del-701	4	4.60 × 10^7^	42/399
		STAT5B	27	6.55 × 10^6^	119/399
		STAT3-β	1	5.98 × 10^6^	120/399
	K562				
		Monobody	517	1.91 × 10^10^	2/578
		STAT3-FL	142	1.31 × 10^7^	39/578
		STAT3-β	2	1.11 × 10^7^	41/578
		STAT3 Del-701	1	1.64 × 10^6^	123/578

Exclusive spectrum counts of monobodies MS3-6 and MS3-N3 and STAT family members upon tandem affinity purification in Jurkat and K562 cells. The complete list of all identified proteins, including keratin contaminants and highly endogenously expressed proteins, is available as a PRIDE database submission: PXD018374.

^a^Including STAT3 isoforms.

^b^All shared peptides between STAT family members and isoforms are associated to STAT3-FL, in addition to its unique peptides. All other STAT family members and isoforms display their unique peptides counts only.

^c^iBAQ values result from the sum of intensities of all tryptic peptides divided by the number of all theoretically observable peptides for each protein. These values provide an accurate determination of the relative abundance of all STATs identified in the samples.

^d^All identified proteins, including common contaminants, such as keratin, were sorted by their iBAQ values from highest to lowest.

Taken together, these results show that MS3-6 and MS3-N3 engage STAT3 as compared to other STAT family members in a highly selective manner in cells without apparent off-targets.

### Monobodies potently inhibit STAT3 transcriptional activity

**Fig. 2:**
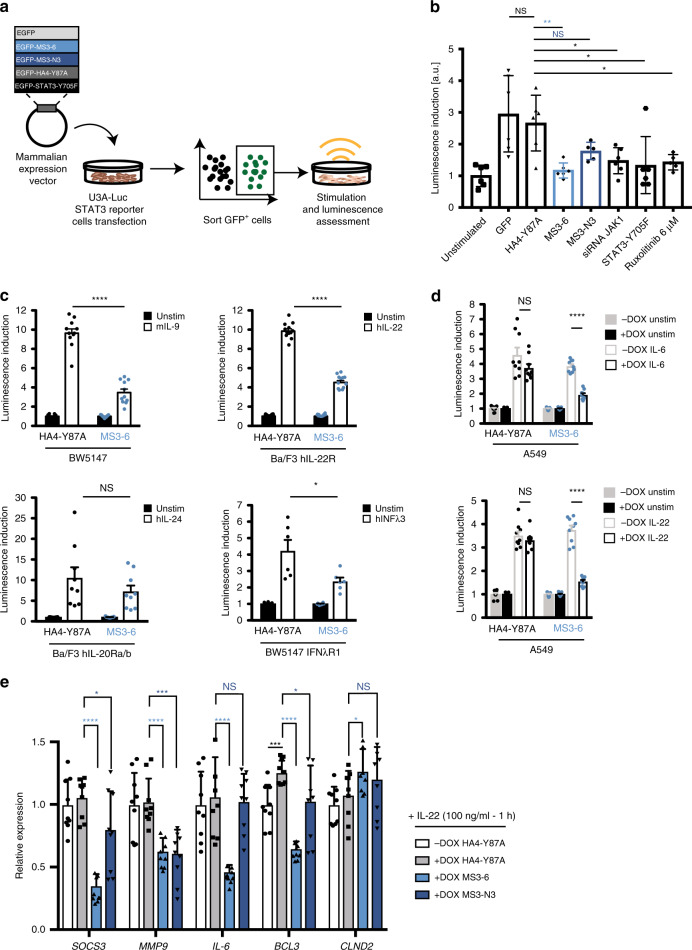
Monobody inhibition of STAT3 transcriptional activity. **a** Scheme depicting the initial screening strategy to identify monobodies with STAT3 inhibitory activity. **b** Luciferase reporter activity inductions normalized to that of unstimulated cells, which was arbitrarily set to 1, are reported for the expression of the indicated monobodies and control treatments. Results from two individual experiments performed in triplicate and/or duplicate are shown (data presented as mean ± SD, *n* = 5 or 6). Two-tailed unpaired *t-*test analysis was performed against the HA4-Y87A monobody control: MS3-6 **P = 0.0027, Ruxolitinib *P = 0.0158, siRNA JAK1 *P = 0.0147, STAT3-Y705F *P = 0.028. **c** BW5147 or Ba/F3 cells (10^7^) were electroporated with the monobody plasmid, the pGL3-Pap1 luciferase reporter plasmid as a specific promoter for STAT3 activation and the pRL-TK plasmid. Cells were stimulated with control medium or murine IL-9 (100 ng ml^−1^, left upper panel, *n* = 11), human IL-22 (500 ng ml^−1^, right upper panel, *n* = 12), human IL-24 (HEK293 supernatant 2%, lower left panel, *n* = 9) or human IFNλ3 (HEK293 supernatant 2%, lower right panel, *n* = 6) for 4 h. Data are presented as mean ± SEM from two to four independent experiments performed in triplicates. **d** Luciferase assay on A549 with inducible monobody (HA4-Y87A or MS3-6) expression. A549 cells (5 × 10^3^) were plated in 96-well plate and treated for 24 h with control medium or doxycycline (1 µg ml^−1^). Cells were transiently transfected with the pGL3-Pap1 luciferase reporter plasmid and the pRL-TK plasmid as internal control of transfection. In all, 4 h after transfection, cells were stimulated with control medium or human IL-6 (100 ng ml^−1^, upper panel) or human IL-22 (500 ng ml^−1^, lower panel) for 20 h. Data are presented as mean ± SEM of three independent experiments performed in triplicates. Significance in **c**, **d** is shown according to two-tailed Mann–Whitney test: *P = 0.026, ****P ≤ 0.0001. **e** RT-qPCR for expression of STAT3 downstream genes in A549 cells expressing monobodies upon IL-22 stimulation (100 ng ml^-1^, 1 hr at 37 °C). Gene expression was normalized to actin and is presented as fold change against that of untreated A549 cells (no monobody expression), which was set to 1. Data from three independent experiments performed in triplicates (*n* = 9) are presented as mean ± SD. Significance was calculated against mRNA levels in the HA4-Y87A monobody control conditions. Significance is shown according to two-tailed unpaired *t-*test analysis: *P ≤ 0.05, **P ≤ 0.01, ***P ≤ 0.001, ****P ≤ 0.0001. Source data are provided as a source data file.

We first tested whether the high-affinity and selective binding of MS3-6 and MS3-N3 interfered with STAT3 cellular functions in a reporter cell line expressing luciferase under the transcriptional control of STAT3 response elements^45^. MS3-6, MS3-N3 and a non-binding monobody control (HA4-Y87A)^44^ were expressed as eGFP fusion proteins, along with eGFP as further negative control, in a doxycycline-inducible expression system. In all, 48 h after the induction of GFP-monobody expression, GFP-expressing cells were FACS-sorted and luciferase activity was measured to infer STAT3 activation upon oncostatin M (OSM) treatment (Fig. 2a). MS3-6 strongly reduced the transcriptional activity of STAT3 as compared to eGFP alone and to the HA4-Y87A monobody control (Fig. 2b). Strikingly, the perturbation of STAT3 activity by MS3-6 were comparable to the effects caused by expression of a transcriptionally inactive dominant negative mutant (STAT3-Y705F), siRNA knock-down of STAT3’s upstream kinase JAK1 or treatment of cells with the potent JAK1/JAK2 kinase inhibitor Ruxolitinib (Fig. 2b). Expression of MS3-N3 inhibited STAT3 transcriptional activity to a lower extent as compared to the positive controls used (Fig. 2b).

As these results demonstrated potent STAT3 inhibition by a monobody, we investigated the activity of MS3-6 in different cellular and stimulatory contexts. We took advantage of BW5147 and Ba/F3 cells that we had engineered to express a panel of cytokine receptors including the IFNλR, the IL-22R and the IL-20Ra/b, known to signal through STAT3^46,47^. Cells were electroporated with plasmids containing either MS3-6 or the negative control HA4-Y87A monobody together with a dual Firefly/Renilla luciferases system under the transcriptional control of STAT3. Stimulation of the respective engineered receptor-expressing cell lines with the mIL-9, hIL-22, hIL24 or hIFNλ3 cytokines strongly induced Firefly/Renilla luciferase signals in the presence of the non-binding monobody control HA4-Y87A (Fig. 2c). In contrast, expression of MS3-6 strongly reduced STAT3 activity upon stimulation by all four cytokines (Fig. 2c). We further tested if MS3-6 inhibits STAT3 transcriptional activity in A549 lung cancer cells, which endogenously express the hIL-6 and hIL-22 receptors. A549 cells were engineered to stably expressed the monobodies using a doxycycline inducible system and then transiently transfected with a STAT3-responsive Firefly/Renilla luciferase reporter. MS3-6 expression strongly decreased STAT3 activity as compared to the HA4-Y87A control following IL-6 and IL-22 stimulation (Fig. 2d), therefore showing that MS3-6 inhibits endogenous STAT3 activation. The inhibitory effect of MS3-6 in the luciferase reporter assays were paralleled by changes in STAT3 target gene expression as analyzed by RT-qPCR in A549 cells. IL-22 stimulation in the presence of MS3-6 severely decreased gene expression of the direct STAT3 target genes SOCS3, MMP9, IL-6 and BCL-3 as compared to the HA4-Y87A monobody control while the effects of MS3-N3 were more modest (Fig. 2e). Interestingly, expression of CLND2 was mildly increased in the presence of MS3-6, but remained unaffected by MS3-N3.

In summary, these data indicate that expression of MS3-6 and, to a lower extend, MS3-N3 potently diminishes the transcriptional activity of STAT3 and the expression of STAT3 downstream target genes.

### MS3-6 is not an SH2-pY inhibitor and decreases DNA binding

**Fig. 3:**
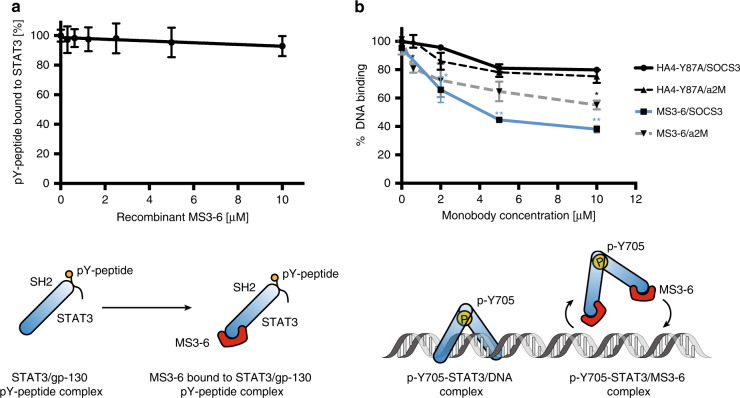
MS3-6 inhibits STAT3 DNA binding, but does not bind the SH2 domain. **a** Addition of increasing recombinant MS3-6 concentrations to a STAT3/phospho-gp130 peptide complex. Fluorescence polarization was measured at the indicated concentration and bound pY-peptide relative to samples in the absence of MS3-6 are plotted. Data from three independent experiments (mean ± SD). **b** Addition of increasing concentrations of recombinant MS3-6 (blue line and grey dashed line) to a pY705-STAT3 dimer bound to fluorescent DNA probes corresponding to downstream promoter sequences (SOCS3 and a2M). Fluorescence polarization was measured at the indicated concentrations of monobodies and bound DNA levels relative to samples in the absence of monobodies are plotted. All fluorescence polarization experiments were performed at 25 °C. Data from two independent experiments (mean ± SD). Significance according to a two-tailed unpaired *t-*test analysis: *P ≤ 0.05, **P ≤ 0.01. Source data are provided as a source data file.

We next surveyed likely mechanisms underlying the molecular action of MS3-6. One way to inhibit STAT3 relies on the blockade of the interaction between its SH2 domain and pY ligands, which perturbs its dimerization and its interaction with upstream cytokines receptors^48^. To investigate whether MS3-6 targets the SH2 domain, we established a fluorescence polarization binding assay with recombinant STAT3-CF and a fluorescent pY-peptide encompassing the pY905 docking site of gp130 (GMPKSpYLPQTVR), the co-receptor of the IL-6 cytokine receptor family. This peptide bound STAT3-CF with a *K*_*D*_ value of 930 nM (Supplementary Fig. 3a). The addition of increasing concentrations of recombinant MS3-6 did not affect binding of the gp130 pY-peptide to STAT3 (Fig. 3a), excluding the possibility that MS3-6 interferes with the SH2-pY ligand interaction.

To assess whether the inhibitory activity of MS3-6 is due to interference with STAT3-DNA binding, we prepared a recombinant tyrosine phosphorylated STAT3 dimer by co-expression of STAT3-CF and the ABL1 tyrosine kinase domain in *E. coli*. Size exclusion chromatography and immunoblot analysis showed dimer formation and strong phosphorylation of Y705 (Supplementary Fig. 3b, c). The purified recombinant STAT3 dimer was assessed for in vitro binding to a fluorescently-labeled double-stranded oligonucleotide corresponding to the STAT3 binding sites in the SOCS3 promoter region, as well as to a higher affinity STAT3 site (a2M)^23^. *K*_D_ values of 1.21 µM and 0.21 µM to the SOCS3 and a2M probes, respectively, were derived from fluorescence polarization binding experiments, in line with previously reported values (Supplementary Fig. 3d)^23^. The addition of recombinant MS3-6, but not of the HA4-Y87A control, decreased binding of the STAT3 dimer to both the SOCS3 and a2M DNA probes in a dose-dependent manner (Fig. 3b). Complete DNA binding inhibition could not be achieved even at high monobody concentration (>1000 fold above *K*_D_). This result thus argues for a contribution of an indirect allosteric inhibition of DNA binding, but cannot explain the strong inhibition of STAT3 signaling by MS3-6 in cells on its own, suggesting the presence an additional mode of action.

### Crystal structure of MS3-6 bound to STAT3

**Fig. 4:**
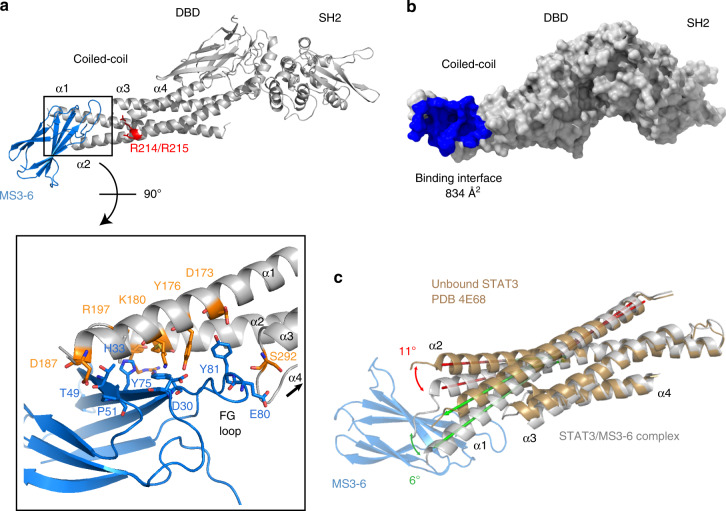
Co-crystal structure of MS3-6 bound to the STAT3. **a** Cartoon representation of the structure of STAT3 (light gray) and monobody MS3-6 (blue). The upper panel shows an overview of MS3-6 binding to the coiled-coil domain of STAT3, with key residues of the nuclear localization sequence (NLS) in close proximity to the monobody binding site highlighted in red. The lower panel shows a magnification of the binding interface with epitope residues (threshold set at 4 Å) depicted as sticks. **b** Surface representation of STAT3 (light gray), with the area covered by the monobody colored in blue. **c** Structural alignment of the coiled-coil domains of STAT3 in complex with MS3-6 together with an unbound STAT3 structure previously published (PDB: 4E68, light brown). Monobody binding leads to a conformational distortion of the helixes α1 and α2. The angles formed between helices are indicated in green and red, respectively.

To understand the mode-of-action of MS3-6 on the molecular functions of STAT3, we solved the crystal structure of MS3-6 in complex with STAT3-CF at a resolution of 2.9 Å (Fig. 4a; Table 3; PDB: 6TLC). The asymmetric unit contains an anti-parallel (unphosphorylated) STAT3 dimer, as previously observed in STAT5A and STAT1 structures (PDB: 1Y1U and 1YVL, respectively). Two monobodies bind one STAT3 dimer, in line with the 1:1 binding stoichiometry determined by ITC (see Fig. 1c). The structure reveals that MS3-6 binds to the helices α1, α2, and to the loop between helices α3 and α4 of the CC domain. Interactions of the monobody with STAT3 are mediated by residues located in its diversified FG loop (a.a. 75–85), together with a diversified position (H33) located in strand βC of the monobody (Fig. 4a; Supplementary Fig 4a.). The overall buried surface area of the STAT3-MS3-6 interface is 834 Å^2^ (Fig. 4b). Protein sequence analysis of all STAT family members showed that most residues implicated in hydrogen bonds or salt bridges with MS3-6 are unique to STAT3 and are not conserved in other STATs (Supplementary Fig. 4b). Additionally, the structure explains the high selectivity of MS3-6 for STAT3 over STAT5, as helix α2 in STAT5 is more than two turns longer and superposition of the structures indicates incompatibility of MS3-6 binding to STAT5 (Supplementary Fig. 4c). Superposition of the MS3-6/STAT3-CF complex and an active STAT3-CF parallel dimer bound to DNA shows that monobody binding does not sterically interfere with DNA binding or pY705-dependent dimerization of the SH2 domains in line with the fluorescence polarization results described above (Supplementary Fig. 4d). Similarly, binding of MS3-6 is compatible with the formation of an anti-parallel unphosphorylated STAT3 dimer (Supplementary Fig. 4e).

**Table 3:** Crystal structure data collection and refinement statistics.

	MS3-6/STAT3-CF
Data collection	
Space group	P 41 21 2
Cell dimensions	
* a*, *b*, *c* (Å)	111.31, 111.31, 483.47
α, β, γ (°)	90.00, 90.00, 90.00
Resolution (Å)	49.78–2.90 (3.1–2.9)^a^
* R*_meas_	34.4 (447.9)
* CC1/2*	99.8 (25.2)
* I* / σ*I*	8.5 (0.61)
Completeness (%)	99.9 (99.8)
Redundancy	14.27 (14.55)
Refinement	
Resolution (Å)	2.9
No. reflections	68566
* R*_work_/*R*_free_	0.246/0.280
No. atoms	
Protein	10063
Water	2
* B*-factors	
Protein	83.24
Water	35.64
R.m.s. deviations	
Bond lengths (Å)	0.015
Bond angles (°)	1.934
Molprobity score	2.61

^a^One crystal per structure. PDB: 6TLC. Values in parenthesis are for the highest resolution shell.

Superposition of the MS3-6/STAT3-CF structure and several previously published structures of STAT3 as well as other STAT family members showed that binding of MS3-6 results in the torsion of the 4-helix bundle (helices α1–4) of the CC and in the bending of the α1 and α2 helices by 4-12 degrees relative to the reference (apo) structures (Fig. 4c and Supplementary Fig. 4f). Hence, MS3-6 binding provokes a conformational change of the CC domain orientation, which may allosterically perturb DNA binding. We furthermore realized that MS3-6 binds close to the nuclear localization signal (NLS) of STAT3, which includes residues R214/215 in helix α2 of the CC domain (Fig. 4a). These two residues are critical for the efficient nuclear import of active phosphorylated STAT3 parallel dimers through binding to importins^26^. Thus, MS3-6 binding may indirectly perturb efficient nuclear import of pY705 phosphorylated STAT3 dimers, leading to a diminished STAT3 transcriptional activity.

In summary, the structural data show that MS3-6 allosterically affects the conformation of STAT3 by altering the CC orientation, thus potentially impairing the nuclear translocation of STAT3.

### MS3-6 reduces STAT3 nuclear translocation upon stimulation

**Fig. 5:**
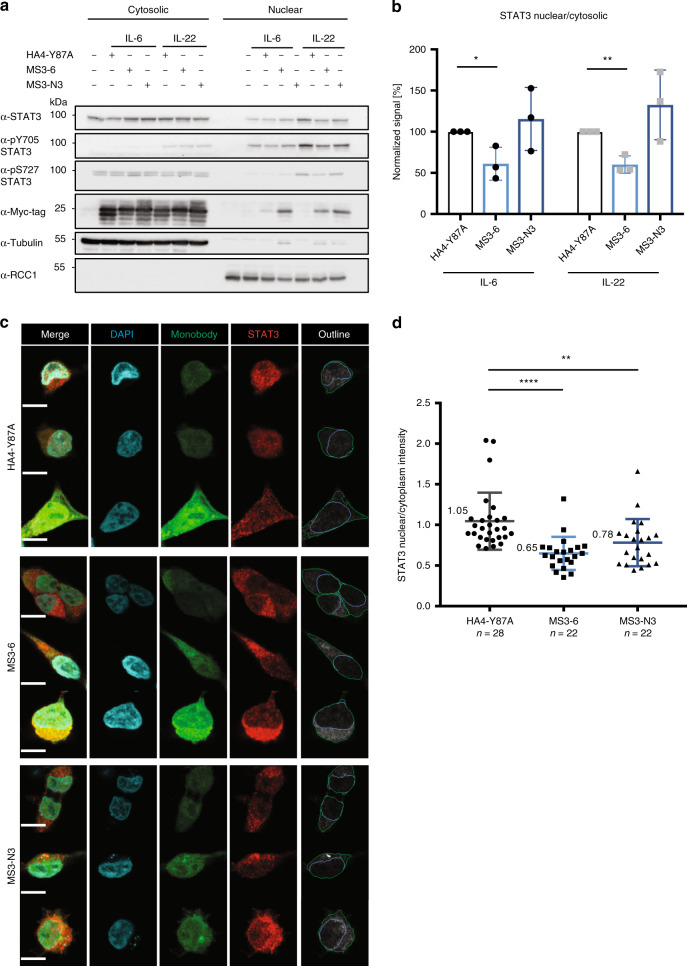
MS3-6 reduces STAT3 nuclear localization. **a** Representative immunoblot analysis of cellular fractionation experiments from three independent experiments. A549 expressing monobodies upon 48 h of 1 µg ml^−1^ doxycycline treatment were stimulated with either IL-6 or IL-22 for 15 min. Nuclear (RCC1) and cytosolic (Tubulin) fractions were recovered and probed for total, p-Y705 and p-S727 STAT3. Quantification of three independent experiments normalized to the HA4-Y87A monobody control are shown in **b** and are plotted as mean ± SD. Significance according to a two-tailed unpaired *t-*test analysis: *P = 0.0278, **P = 0.0028. **c** Confocal microscopy images from two independent experiments of HEK293 cells expressing a monobody-GFP fusion and treated with IL-6 to assess nuclear translocation. Scale bar represents 10 µm. The right column shows the outlines used to determine nuclear and cellular compartments indicated by blue and green lines, respectively, using the CellProfiler software. The number of individual cells analyzed (*n*) is indicated on the figure. Quantification of STAT3 nuclear/cytoplasmic levels from two independent experiments is shown in **d**. Significance according to a two-tailed unpaired *t-*test analysis: **P = 0.0065, ****P ≤ 0.0001. The number of analyzed cells is indicated below the graph. Source data are provided as a source data file.

The close proximity between the monobody binding site and the NLS led us to hypothesize that MS3-6 impairs STAT3 nuclear translocation. To test this model, we performed subcellular fractionation and immunofluorescence experiments. The levels of STAT3 in the nuclear and cytoplasmic fractions were determined by immunoblot in A549 cells stimulated with IL-6 or IL-22 in the presence of MS3-6, MS3-N3 or the negative control HA4-Y87A monobody. MS3-6 led to a reduced level of nuclear STAT3 upon stimulation with either IL-6 or IL-22 (Fig. 5a). Similarly, MS3-6 decreased the nuclear:cytosolic-ratio of STAT3 and pY705-STAT3 (Fig. 5b and Supplementary Fig. 5a, b) as well as the STAT3:RCC1- and pY705-STAT3:RCC1-ratio used to correct for different fractionation efficiencies (Supplementary Fig. 5c). MS3-N3 failed to inhibit nuclear translocation of STAT3, in line with the distinct binding site of this monobody in the N-terminal domain, which is not in close proximity to the NLS. Therefore, in order to further evaluate the impact of monobodies on nuclear translocation, we additionally took advantage of confocal immunofluorescence microscopy using HEK293 cells inducibly expressing GFP-monobody fusions upon doxycycline treatment, which were stimulated with IL-6. Again, we observed reduced nuclear STAT3 levels in cells that expressed MS3-6 as compared to cells expressing HA4-Y87A (Fig. 5c and Supplementary Fig. 5d). Quantitative image analysis corroborated these qualitative results (Fig. 5d).

Altogether, these data determine that the inhibition of the transcriptional activity of STAT3 by MS3-6 is mediated by reduced STAT3 nuclear translocation.

### MS3-6 reduces STAT3 Y705 phosphorylation in IL-22R signaling

**Fig. 6:**
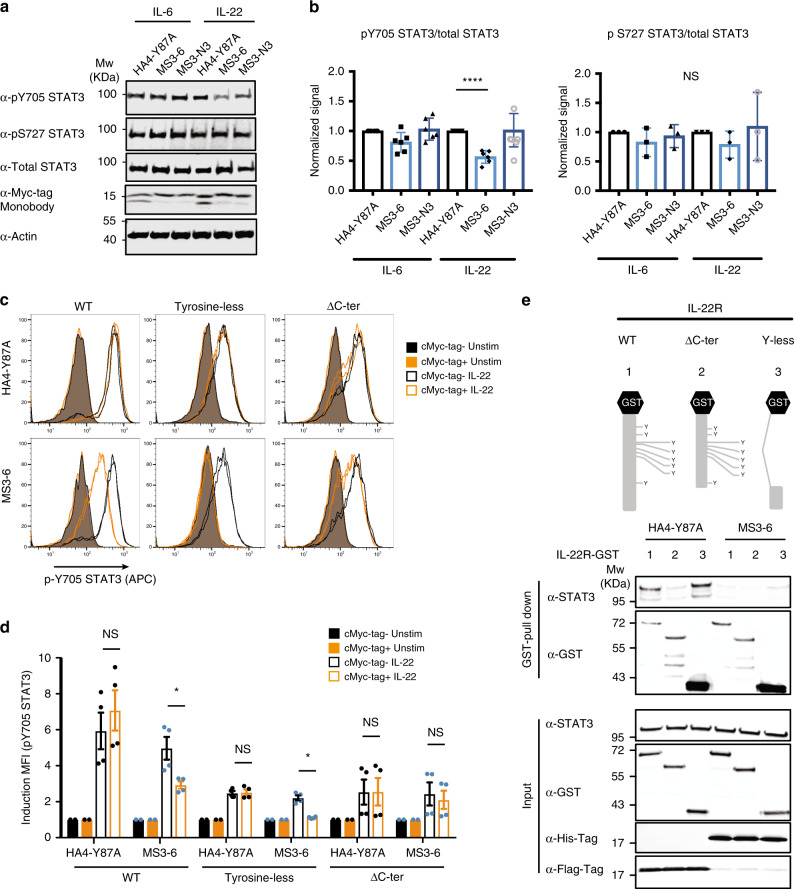
MS3-6 reduces STAT3 Y705 phosphorylation levels upon IL-22 stimulation. **a** Representative immunoblot from five (pY705) and three (pS727) independent experiments of STAT3 phosphorylation levels following IL-6 and IL-22 stimulation for 15 min. All immunoblots are available as Source data Files. **b** Quantification (mean ± SD) of the results in **a** from all independent experiments for the indicated STAT3 phosphorylation sites. Significance according to an unpaired two-tailed *t-*test analysis: ****P ≤ 0.0001. **c** Flow cytometry analysis on Ba/F3 cells expressing IL-22R. Four hours after electroporation of the monobody vectors (15 µg), cells were stimulated with IL-22 (500 ng ml^−1^) and staining was performed. A live-cell gating strategy was applied and phospho-STAT3 staining was analyzed in cMyc-tag^+^ and cMyc-tag^−^ cells. Unstimulated cells are reported as colored bar plot and stimulated cells are reported as empty bar plots. **d** Quantification of pY705-STAT3 staining from **c**. Data are presented as mean ± SEM of two independent experiments performed in duplicates (*n* = 4). Significance according to a two-tailed Mann–Whitney test analysis: *P ≤ 0.05. **e** Upper panel, schematic representation of GST fusion proteins with intracytoplasmic domain of IL-22R. Lower panel, COS-7 cells were seeded in six-well plate and transfected with a vector coding for the GST-fusion protein or STAT3. Cells were lysed and the recovered STAT3 protein was mixed with the recombinant monobody (10 µM) before incubation with IL-22R-GST overnight. Proteins eluted on GST SpinTrap columns as well as input samples were analyzed by western blot with an anti-STAT3 antibody. Membrane was then re-probed with anti-GST and anti-tag antibodies. Source data are provided as a source data file.

To investigate the effects of the monobodies on endogenous STAT3 signaling, we inducibly expressed monobodies in A549 lung cancer cells. Expression of MS3-6, MS3-N3 or the HA4-Y87A control did neither strongly impact STAT1 tyrosine phosphorylation nor STAT3 serine 727 phosphorylation (Fig. 6a, b and Supplementary Fig. 6a). Interestingly, MS3-6 led to an increased STAT5 phosphorylation upon IL-22 stimulation (Supplementary Fig. 6a, b). While MS3-6 induction had no significant effect on pY705 STAT3 upon IL-6 stimulation, we observed a strong reduction of Y705 phosphorylation following IL-22 stimulation by immunoblotting (Fig. 6a, b). This unexpected finding was confirmed by FACS analysis. We again detected reduced pY705 STAT3 levels upon IL-22 stimulation, but not following IL-6 stimulation (Supplementary Fig. 6c–g). We reasoned that different molecular mechanisms of STAT3 activation by the two cytokine receptors may account for the observed difference, as IL-22 stimulation was reported to utilize a non-canonical constitutive association of STAT3 with the IL-22 receptor in the absence of cytokines^27^. This alternative STAT3 stimulation is mediated by the constitutive interaction between the CC of STAT3 and the 84 C-terminal amino acids of the IL-22R, which lack tyrosine residues. IL-22-dependent STAT3 activation can be triggered by a phospho-tyrosine independent mechanism in addition to the conventional (phospho-tyrosine dependent) recruitment of STAT3 to the IL-22R following stimulation.

To determine if the CC-binding monobody MS3-6 perturbs IL-22 signaling, we used Ba/F3 cell lines expressing IL-22R mutants, which include a tyrosine-less mutant where all tyrosine residues located in the intracellular domain of IL-22R were mutated to phenylalanine, and a C-terminal truncation (IL-22R receptor lacking the last 84 amino acids; ∆C-ter). These IL-22R mutants allowed us to dissect the impact of the MS3-6 monobody on conventional (∆C-ter mutant) and alternative (tyrosine-less mutant) STAT3 activation. The Y705 phosphorylation status of STAT3 was assessed in cells expressing the different IL-22R mutants by flow cytometry following IL-22 stimulation upon electroporation of the Myc tagged monobody plasmids. MS3-6 strongly decreased STAT3 phosphorylation after IL-22 stimulation in cells expressing the wild-type and tyrosine-less receptor, but not the ∆C-ter receptor, highlighting the impact of the monobody on the non-canonical IL-22 signaling pathway (Fig. 6c, d). To further corroborate this result, we performed GST-receptors pull-down experiments. STAT3 was robustly co-purified with the recombinant GST-IL-22R intracellular domain mutants in cells that expressed the control HA4-Y87A monobody, as long as the IL-22R contained its C-terminal part (Fig. 6e). In contrast, expression of MS3-6 resulted in the loss of STAT3 co-purification.

These results reveal that MS3-6 prevents the binding of STAT3 to the C-terminus of IL-22R, which indicates selective perturbation of alternative non-canonical IL-22 mediated STAT3 activation.

## Discussion

Transcription factors are essential players in the development of pathological conditions such as cancer^49^. Nonetheless, targeting transcription factors is very challenging due to the lack of deep pockets able to fit an antagonistic small molecule inhibitor^50^. The inhibition of STAT3 in cancer is highly desirable^51,52^ and a lot of effort is invested towards the therapeutic inhibition of STAT3 in academia and the pharmaceutical industry. Still, there are no FDA-approved drugs targeting STAT3 directly yet. Current efforts focus on inhibiting STAT3-dimerization by developing antagonists of the SH2 domain^19^, whereas targeting additional domains that are important for pathological STAT3 signaling remain poorly studied. In this work, we establish monobodies as tools to antagonize the activity of a transcription factor. Our results indicate that both MS3-6 and MS3-N3 bind to STAT3 with low nanomolar affinities in both yeast binding and ITC assays, but the former assay consistently resulted in lower apparent binding affinities. Given that yeast display is not a true equilibrium binding assay, these differences are not surprising. Thus, while yeast display is less laborious, as no purified high-quality monobody protein is required, a true equilibrium binding assay, such as ITC, is indispensable to precisely determine *K*_*D*_ values of lead candidate monobodies.

In contrast to the monobodies previously developed against several other SH2 domain-containing oncogenes^33,35,37^, none of the tested STAT3 monobodies target the SH2 domain, but instead bind the CC domain and inhibit transcriptional activity of STAT3. In a separate selection, monobodies targeting the NTD were developed. The NTD has been shown to fine tune transcriptional responses of STAT3 by favoring the formation of STAT3 tetramers, which allow the recognition of weaker DNA binding sites and regulation of chromatin organization^13,22,53^. Similarly, the NTD of STAT3 was found to repress the expression of pro-apoptotic genes^23,54^. The use of MS3-N3 to study NTD functions may prove as a very valuable tool to understand STAT3 biology in the future. Because the specific blockade of the NTD using MS3-N3 monobody was found to interfere to a lower extend with the transcriptional activity of STAT3, we concentrated our efforts on the characterization of MS3-6 in this study. Therefore, the mode of action of MS3-N3 remains to be studied in more detail, as part of an independent study in the future. In particular, the differential regulation of STAT3 target gene expression by MS3-6 and MS3-N3 may hint towards interesting underlying biology requiring a more in-depth study. Likewise, the observed trend of an inhibitory activity of MS3-N3 on IL-6 induced nuclear translocation of STAT3 should be followed-up.

**Fig. 7:**
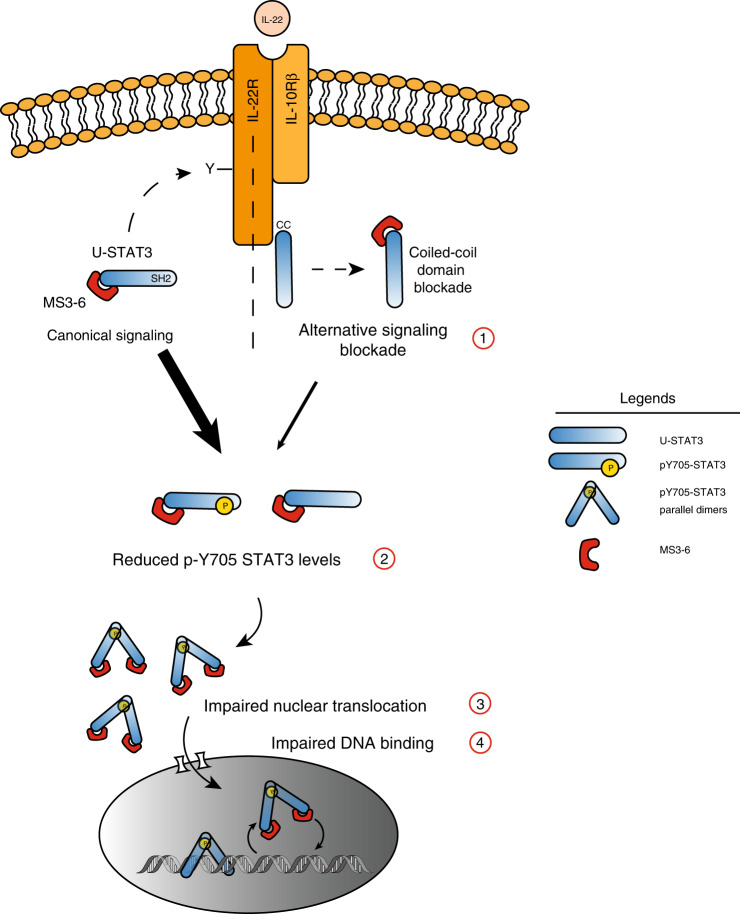
Model of the mode-of-action of MS3-6. The cumulative influences (labeled 1–4) of MS3-6 binding on inhibition of STAT3 transcriptional activity is schematically illustrated based on the presented results. MS3-6 decreases STAT3 Y705 phosphorylation levels by impairing the binding of STAT3 to the IL-22R cytosolic tail, thus specifically blocking the alternative IL-22R/STAT3 signaling axis (1) and (2). Additionally, MS3-6 leads to the reduction of STAT3 translocation in the nucleus upon cytokine stimulation (3) and decreases STAT3 binding to DNA (4). Legend: U-STAT3: unphosphorylated STAT3.

We showed binding of MS3-6 in proximity to a proposed NLS including residues R214/R215^26^ and inhibition of nuclear translocation. It is therefore not unlikely that MS3-6 interferes with importin-α3 binding to this NLS. The lack of a complete inhibition of nuclear translocation can possibly be explained by the presence of a second NLS in the DBD. This NLS includes residues R414/R417, which are recognized by importin-α5 and -α7^24^ and whose accessibility is predicted to be not affected by MS3-6 binding. Therefore, the relative contributions of the two NLSs to overall STAT3 nuclear translocation will determine the severity of MS3-6 perturbation. In summary, our data indicate that monobody MS3-6 interferes at various levels to inhibit STAT3 phosphorylation, reduce its nuclear translocation and diminish DNA binding. Hence, our results suggest that the inhibitory mode of action of MS3-6 relies on a cumulation of distinct mechanisms acting in synergy towards the overall reduction of STAT3 transcriptional activity (Fig. 7).

A strong limitation of many targeted therapies is off-target toxicity, in particular when protein classes with hundred and more homologous members, such as protein kinases or transcription factors are targeted. In contrast, our monobodies were found to be highly selective for STAT3 in different cellular proteomic contexts. While structural modeling indicated that the different lengths of helices in STAT1 and STAT5 would hinder MS3-6 binding, the residual amounts of STAT1 and STAT5 in the TAP-MS experiments can be explained by the ability of STAT3 to form heterodimers with other STATs. The preferential binding of our monobodies to STAT3 may also prove useful for combining inhibition of STAT3 signaling with targeted degradation approaches, as exemplified by the degradation of STAT3 upon expression of monobody-VHL fusions. The distinct degradation efficiencies between MS3-6 and MS3-N3 may be explained by their differences in affinity for STAT3 or by the positioning of lysine residues for ubiquitylation in proximity of the monobody binding site. Altogether, our work highlights how the rapid development of high affinity protein binders, such as monobodies, appear particularly suited for a targeted degradation strategy to mediate the efficient suppression of undruggable target proteins for which we currently lack small molecule binders.

Future efforts in validating the use of these monobodies as protein-based therapeutics in physiological systems should aim at exploring delivery strategies for therapeutic approaches and in vivo applicability. Importantly, our study shows that in addition to the inhibition of the transcriptional activity of STAT3, the specific blockade of the CC domain with MS3-6 provided additional insights in a poorly characterized cytokine signaling pathway, namely into alternative IL-22R signaling in which STAT3 is pre-associated via its CC domain to the IL-22R in the absence of cytokine. Therefore, our study highlights the role of monobodies as molecular tools to dissect poorly understood signaling pathways. Lastly, our study provides a proof of concept towards the inhibition of transcription factors implicated in human cancer and other human diseases using monobody inhibitors and lays the foundations for the targeting of additional transcription factors currently regarded as undruggable.

## Methods

### Antibodies, cell lines, and reagents

Antibodies against STAT1 (No. 9172), pY701 STAT1 (No. 9167), STAT2 (No. 4597), STAT3 (No. 9139), pY705 STAT3 (No. 9145), pS727 STAT3 (No. 9134), STAT4 (No. 2653) and pY694/pY699 STAT5 (No. 9359) were purchased from Cell Signaling Technology. The antibody against STAT5B (sc-1656) was obtained from Santa Cruz Biotechnology. The ubiquitin antibody (No. 04–263, clone FK2) was purchased from Merk. The anti-Myc tag antibody directly coupled to Dylight800 was purchased from ThermoFisher (No. MA1-21316-D800). The antibody against penta-His was ordered from Qiagen (No. 34610) and the anti-alpha Tubulin antibody (T9026) was ordered from Sigma-Aldrich. All primary antibodies were used at a 1:1000 dilution in 5% BSA TBS-T solution or 5% milk TBS-T according to manufacturer instructions. Secondary antibodies used in western blots were obtained from LiCOR: anti mouse IRDye680 (No. 926–68070), anti-mouse IRDye800 (No. 926–32210), and anti-rabbit IRDye680 (No. 925–68071). Similarly, the secondary anti-mouse or anti-rabbit HRP coupled antibodies were purchased from Cell Signaling Technology (No. 7076 and 7074 respectively). Streptavidin Magnesphere Paramagnetic Particles (Promega #Z5481) were used for the recombinant biotinylated protein pull-down and during the monobody selection. Ba/F3, K562 and Jurkat cells were purchased from DSMZ (Deutsche Sammlung von Mikroorganismen und Zellkulturen, Braunschweig, Germany, Cat# ACC-300, ACC-10 and ACC-282, respectively). A549 cells were a kind gift from E. Meylan (EPFL). NPM-ALK cells were established from mouse thymoma in the lab of V. Sexl (Vet Med Uni Wien). Hek293 cells were obtained from ATCC (Cat# CRL-1573) and U3A-luc cells were a kind gift from David A. Frank (Harvard). The cytokines used in this work, human Oncostatin M (O9635) and IL-22 (SRP3089) as well as Human IL-6 (MAN0003501) were purchased from Sigma-Aldrich and Gibco, respectively.

### Plasmids and cloning

Human STAT3 cDNA was purchased from the Gene Expression Core Facility at EPFL. The NTD [aa 3–138] and core fragment of STAT3 [aa 129–722] were cloned using the restriction enzymes BamHI and XhoI into a pHBT vector^35^ comprising an N-terminal 6xHis tag, Avi-tag for biotinylation and tobacco etch virus (TEV) protease cleavage site. For lentiviral transduction, monobodies and VHL-monobodies fusions were cloned into an inducible pEM24 vector (modified pCW2239 obtained from E. Meylan, EPFL) using InFusion recombinase (Clontech). The lentiviral expression system vectors pCMV-R8_74 (encoding gag and pol proteins) and pMD2_G (encoding VSV-G envelope) were obtained from the Trono Lab at EPFL. Retroviral transduction was performed using a constitutive pRV-NTAP vector containing a 2xProteinG-TEV-cMyc tag. Monobodies were inserted in the pRV-NTAP vector using getaway cloning. Retroviral expression system encoding the VSV-G envelope (pCMV-VSV-G) was obtained from the Superti-Furga lab. Monobody-GFP fusions were introduced into a doxycycline inducible pEBtetD vector using gateway cloning. Similarly, monobodies were inserted into a pCS2 N-term 6x-Myc tag vector using gateway cloning. All DNA constructs were confirmed by DNA sequencing (Microsynth). A complete list of primers and description of vectors used in this work is provided in Supplementary Tables 1–4.

### Monobody selection

Biotinylation of Avi-tagged proteins targets (STAT3) was performed in vivo by co-transforming *Escherichia coli* cells with a BirA plasmid and a pHBT plasmid containing the AviTag gene fusion. Cells were induced with 0.5 mM of IPTG once the OD600 of the expression culture medium between 0.5 and 0–8. A final Biotin concentration of 50 μM from a 50 mM stock in DMSO was added. Cells were further grown overnight before purification. Monobodies were selected according to methods previously described^31,55^. Briefly, four rounds of phage display were followed by the amplification and transformation of yeast cells with the DNA sequences corresponding to binding monobodies. The yeast cells were next sorted using FACS based on a strict gating strategy comprising double positive cells for monobody display and binding to biotinylated target. Isolated clones were next sequenced and cloned into pHBT vectors for further characterization.

### Yeast binding assay

Increasing concentrations of recombinant biotinylated protein targets resuspended in Tris-buffered saline (TBS) with 0.1% BSA were incubated with yeast cells displaying monobodies and a mouse anti-V5 antibody for 30 min at room temperature. Three washes with TBS with 0.1% BSA followed together with incubation for 30 min with a streptavidin-DyLight650 and a FITC-coupled anti-mouse IgG at room temperature in the dark. Samples were analyzed on a Gallios flow cytometer (Beckman Coulter). Data were fitted on a 1:1 binding model using the Prism software (Graphpad 7) to determine K_D_ values.

### Isothermal titration calorimetry

Recombinant proteins were dialyzed overnight at 4 °C in Isothermal titration calorimetry (ITC) buffer (25 mM Hepes (pH 7.5) and 150 mM NaCl) and were degassed. Total protein concentration was measured using the 280 nm protein absorbance. ITC measurement were acquired on a MicroCalPEAQ (Malvern panalytical) instrument and consisted in the titration of the protein solution from the syringe in 16 steps with 0.49 μL for the first injection followed by 2.49 μL for the remaining steps. Protein concentrations used were 100 µM (syringe) titrated to a 10 µM solution (cell) as indicated on the figure legends. The MicroCal software was used to determine thermodynamic parameters.

### Quantitative reverse transcription-polymerase chain reaction

Total RNA from A549 cells expressing the monobodies for 48 h followed by IL-22 (100 ng ml^−1^) stimulation (1 h at 37 °C) was isolated using TRIzol reagent (Ambion, life technologies No. 15596–026). Primers for known STAT3 downstream target genes were obtained from the Etienne Meylan lab (EPFL) and were originally purchased from ThermoFisher (TaqMan Assays). Primer sequence references (cat# 4331182) are as follow: SOCS3 (Hs02330328_s1), BCL3 (Hs00180403_m1), MMP9 (Hs00234579_M1), IL-6 (Hs00985639_m1), Cldn2 (Hs00252666_s1), Actin (Hs01060665_g1) and 18S (Hs99999901_s1). Each PCR reaction contained 1 µl of cDNA (from a RT-PCR reaction using 1000 ng of total RNA) and 0.5 µl of each 10 µM primer mix. PCR conditions consisted in 95 °C for 5 min followed by 35 cycles of 30 s at 60 °C. Actin housekeeping gene was used for normalization of signals. All Quantitative reverse transcription-polymerase chain reaction (RT-qPCR) analyses were performed in triplicates from three independent experiments.

### Recombinant protein expression and X-ray crystallography

BL21 (DE3) E. coli cells were transformed with pHBT-monobody and pHBT-STAT3-CF constructs. An overnight pre culture was added to LB medium with an original OD at 0.05–0.1. Cells were grown at 37°, 200 rpm until OD reached 0.5–0.8 and the culture was next induced with 0.5 mM IPTG final concentration. Cells were further grown overnight at 18 °C 200 rpm. The next day, cells were harvested at 6000 g for 10 min at 4 °C and resuspended in 40 ml/L of culture in Buffer A [25 mM Tris (pH 7.5), 300 mM NaCl, 5% glycerol, and depending on sample, 1 mM DTT]. Cells were lysed using an Avestin Emulsiflex homogenizer and the soluble proteins were isolated from the cell debris by centrifugation at 41,000 g for 30 min at 4 °C. Proteins of interest were next purified by affinity using a Ni-NTA resin and size-exclusion chromatography using a Superdex 200 16/600 GL column. Complex formation was performed by mixing TEV cleaved-MS3-6 and STAT3-CF in a 1:1 molar ratio into gel filtration buffer (20 mM HEPES pH 7, 200 mM NaCl, 10 mM MgCl2 and 2 mM DTT). The complex was concentrated in Amicon vivaspin tubes to 11 mg/ml and 0.6 mM dsDNA (forward: TGCATTTCCCGTAAATCT3′ and reverse: 5′AAGATTTACGGGAAATGC3′) was added to the drop before crystallization trials in hanging drop plates by mixing 100 nL of protein with 100 nL of buffer conditions. Addition of DNA was necessary for crystallization, despite not appearing in the final structure. Crystals were optimized in a 24-well plate format (hanging drop) by mixing 1 μL of protein with 1 μL of buffer. The best diffracting crystals were obtained in 19% PEG300, 70 mM Calcium acetate dihydrate, 100 mM imidazole pH 7 conditions, frozen in 15% (v:v) glycerol in liquid nitrogen. Data were collected at the beam-line PXIII of the Swiss Light Source (SLS), Villigen, Switzerland. Data were collected at 1 Å and at 100 K. Data was processed in XDS^56^. The structure was determined by the molecular replacement method (molrep, PHENIX version 1.1.17) using a previously determined STAT3 structure (PDB:4E68) and a homology model of the monobody MS3-6 as initial search models. Manual model building of the structure was performed using Phaser and Coot and refined with REFMAC^57^. The final model geometry was with 93.2% in favored regions of the Ramachandran plot with 1.8% of Ramachandran outliers.

### Tandem affinity purification and mass spectrometry

Monobodies cloned into the retroviral TAP tagged vector were used to establish Jurkat and K562 stable cell lines. Cells expressing the IRES-GFP were selected and sorted using FACS. The TAP purification consisted in the lysis of used 2–3 × 10^9^ cells as previously described^58^. Briefly, the elution of the protein complexes following two steps of affinity purification was performed using 0.1 M hydrochloric acid immediately followed by the neutralization using 0.5 M Triethyl ammonium bicarbonate. Samples were then boiled and 10% of the eluates were resolved by SDS-PAGE (4–20% gel; Bio-rad) and silver stained to assess the efficiency of the pull-down. The rest of the eluates were then separated by SDS-PAGE and stained with R-250 Coomassie Blue Solution. For all single biological experiments, the entire SDS-PAGE gel lanes were first sliced into 4 fractions resulting in a total of 16 samples, which were washed twice in 50% ethanol and 50 mM ammonium bicarbonate (AB, Sigma-Aldrich) for 20 min and dried by vacuum centrifugation. Sample reduction was performed with 10 mM dithioerythritol (Merck-Millipore) for 1 h at 56 °C. A washing-drying step as described above was repeated before the alkylation step performed with 55 mM Iodoacetamide (Sigma-Aldrich) for 45 min at 37 °C in the dark. Samples were washed-dried again and digested overnight at 37 °C using modified mass spectrometry grade trypsin (Trypsin Gold, Promega) at a concentration of 12.5 ng µl^−1^ in 50 mM AB and 10 mM CaCl_2_. Resulting peptides were extracted in 70% ethanol, 5% formic acid (FA, Merck-Millipore) twice for 20 min with permanent shaking. Samples were further dried by vacuum centrifugation and stored at −20 °C. Peptides were desalted on StageTips^59^ and dried under a vacuum concentrator prior to LC-MS/MS injection. Samples were resuspended in 2% acetonitrile (Biosolve), 0.1% FA and nano-flow separations were performed on a Dionex Ultimate 3000 RSLC nano UPLC system (Thermo Fischer Scientific) on-line connected with a Q Exactive Orbitrap Mass Spectrometer (Thermo Fischer Scientific). Acquisitions were performed using Data-Dependent acquisition mode. First MS scans were acquired with a resolution of 70,000 (at 200 m/z) and the 12 most intense parent ions were selected and fragmented by High energy Collision Dissociation (HCD) with a Normalized Collision Energy (NCE) of 27% using an isolation window of 1.2 m/z. Fragmented ions were acquired with a resolution 17’500 (at 200 m/z) and then excluded for the following 30 s. A capillary precolumn (Acclaim Pepmap C18, 3 μm-100 Å, 2 cm × 75 μm ID) was used for sample trapping and cleaning. A 50 cm long capillary column (75 μm ID; in-house packed using ReproSil-Pur C18-AQ 1.9 μm silica beads; Dr. Maisch) was then used for analytical separations at 250 nl min^−1^ over 150 min. biphasic gradients. Raw data were processed using SEQUEST (precursor and fragment ion mass tolerances were 10 ppm and 0.05 Da, respectively) in Proteome Discoverer v.2.2 against a concatenated database consisting of the Uniprot human protein database (95796 entries) and monobodies sequences. Enzyme specificity was set to Trypsin and a minimum of six amino acids was required for peptide identification. Up to two missed cleavages were allowed. A cut-off was fixed at 1% FDR at the peptide and protein identification level. For the interactome database search carbamidomethylation was set as a fixed modification, whereas oxidation (M), acetylation (protein N-term), PyroGlu (N-term Q), were considered as variable modifications. Data was further processed and inspected in Scaffold 4.10 (Proteome Software, Portland, USA) and spectra of interest were manually validated. In order to analyze data in an unbiased way and filter for most unspecific protein contaminants, a selectivity score was calculated as a function of the number of TAP experiments similarly performed in Jurkat and K562 cells using unrelated monobodies (including the HA4-Y87A control)^35,37,44,60^ a given protein was detected in, as compared to the total number of TAP experiments we have performed and previously published. A threshold of >75% was applied to determine the likelihood of a protein being a specific monobody interactor. This scoring permits the identification of proteins found in only <25% of all the TAPs in our internal database, thus allowing most unspecific contaminating or highly expressed cytosolic proteins recovered from the pull down to be filtered out. Common contaminants and unspecific proteins identified were included in protein ranking shown in Table 2 for transparency. All mass spectrometry proteomics data from TAPs have been deposited to the ProteomeXchange Consortium via the PRIDE^61^ partner repository with the dataset identifier PXD018374.

### Western blot analysis

STAT3 phosphorylation in A549 cells was assessed upon treatment with IL-6 or IL-22 (500 ng ml^−1^) for 15 minutes at 37 °C. Total protein extraction was done in lysis buffer (50 mM Tris pH 8, 150 mM NaCl, 1% Triton X-100) supplemented with 50 mM NaF, 1 mM Vanadate, 1 mM PMSF, 10 µg ml^−1^ TPCK and protease and phosphatase inhibitors (Roche). Protein concentration was measured using a Bradford assay (Bio-Rad No. 500–006) or BCA (Thermo scientific No. 23225) and equal amounts of proteins were loaded on a SDS-polyacrylamide electrophoresis (PAGE) gel. Transfer to nitrocellulose membranes was performed using the semi-dry (Bio-Rad) blotting system or to PVDF membranes by overnight wet transfer. Membranes were next incubated overnight at 4 °C with primary antibodies followed by 1 h room temperature incubation with secondary antibodies. Fluorescent or chemiluminescent detection was performed using the LiCOR and Bio-Rad ChemiDoc Imaging systems. Protein expression levels were quantified using the ImageStudio or Image Lab software and their relative amounts with respect to tubulin were calculated. All blots were performed in three independent experiments. All uncropped western blots are provided in the source data file.

### Nuclear/cytoplasmic fractionation and immunofluorescence

Nuclear fractionation was performed upon stimulation of A549 cells for 15 minutes at 37 °C with 100 ng ml^−1^ of IL-6 or IL-22. Cytosolic fractions were recovered by lysing cells in cytosolic buffer (10 mM HEPES pH 7.9, 10 mM KCl, 0.1 mM EDTA, 0.1 mM EGTA supplemented with 2 mM DTT, 0,4 mM Na-Vanadate, 25 mM Na-Fluoride,1 mM PMSF, 20 µg ml^−1^ Leupeptin and 20 U ml^−1^ Aprotinin). Extensive washing in cold PBS followed (resuspended five times in 1 ml, for 5 min each), and the nuclear pellet was recovered by centrifugation (13,000 g at 4 °C) and lysed in nuclear buffer (20 mM HEPES pH 7.9, 25% Glycerol, 400 mM NaCl, 1 mM EDTA and 1 mM EGTA supplemented with 2 mM DTT, 0,4 mM Na-Vanadate, 25 mM Na-Fluoride, 1 mM PMSF, 20 µg ml^−1^ Leupeptin and 20U ml^−1^ Aprotinin). Western blot analysis was performed as described above.

Immunofluorescence was performed using transiently transfected HEK293 cells (Polyfect Qiagen No. 301107) that expressed an inducible GFP-tagged monobody upon doxycycline treatment (1 µg ml^−1^) for 48 h. Cells were stimulated with IL-6 (100 ng ml^−1^) for 20 minutes at 37 °C and were fixed in 4% paraformaldehyde (PFA) for 15 minutes at room temperature. Slides were next washed with PBS containing 0.01% of Triton-X and stained for 1 h at room temperature using a primary antibody (mouse anti-STAT3). Secondary antibody detection was performed using an anti-mouse Alexa Fluor 568 antibody. Hoechst staining was performed for 5 min during the last wash. Cells were imaged with a Zeiss LSM 700 confocal microscope. Image analysis and cellular compartments were defined according to a CellProfiler workflow: a primary object detection defined the nucleus according to the Hoechst staining. Secondary object detection was performed by expansion of the GFP signal around the nucleus, which defined the cell outlines. The tertiary object corresponded to the nucleus area removed from the cell outline, which defined the cytoplasm. Mean intensity of both GFP and the anti-mouse Alexa Fluor 568 secondary antibody signals (STAT3) were quantified in all objects. Data were presented as a ratio of nuclear STAT3:cytoplasm STAT3. A total of approximately 30 cells from two individual experiments were acquired and quantified. Cells whose automatic boundary detection failed were discarded from final analysis.

### Fluorescence polarization

A pY-peptide corresponding to the cytosolic tail of the gp130-Y905 co-receptor was used (5FLU-GMPKSpYLPQTVR-NH_2_). A 50 µl solution consisting in 250 nM of the peptide mixed with 1.25 µM of recombinant STAT3-CF followed by an addition of increasing concentrations (0–10 µM) of recombinant MS3-6 in TBS buffer (50 mM Tris pH 7.5 and 150 mM NaCl) was prepared. Measurements were performed in FP compatible dark bottom 96-well plates at room temperature. Wavelength set for data acquisition was 525 nm with a filter at 515 nm. Data were obtained using a M5 plate reader from Molecular Devices. Raw data were normalized to free peptide in TBS solution as background measurement.

Similarly, two DNA oligonucleotides sequences corresponding to STAT3 binding sites were used: SOCS3 (forward: 5′FAM-GCAGTTCCAGGAATCGG3′/reverse: 5′CGTCAAGGTCCTTAGCC3′) and a2M (forward: 5′FAM-AGCAGTAACTGGAAAGTCCTTAATCCTTCTGGGAATTCT3′/reverse: 5′AGAATTCCCAGAAGGATTAAGGACTTTCCAGTTACTGCT5′). Oligos were initially mixed at equimolar concentration in annealing buffer (10 mM Tris pH 7.5, 50 mM NaCl and 1 mM EDTA) and annealed by PCR (95 °C for 2 min, then slowly decrease to RT). Once double-stranded DNA was obtained, a solution consisting of 25 µl of 10 nM dsDNA probes were added to 12.5 µl of 2 µM recombinant p-STAT3 dimer and 12.5 µl of increasing MS3-6 concentrations (final concentrations: 0, 0.5, 1, 2, 5, and 10 µM). The final 50 µl solution was seeded in FP compatible dark bottom 96-well plates. Fluorescence polarization measurements were performed at room temperature. Wavelength set for data acquisition was 485 nm with a filter at 528 nm. Data were obtained using a M5 plate reader from Molecular Devices. Raw data were normalized to free DNA in solution as background measurement.

### GST pull down assay

To monitor the interaction between the different GST-IL-22R mutants generated previously^27^ and STAT3, the proteins were expressed independently in COS-7 cells. Briefly, 4 × 10^5^ COS-7 cells plated in a six-well plate were transiently transfected using ViaFect (Promega), according to the manufacturer’s recommendations. Two days later, cells were lysed in 500 µl of lysis buffer (1% Triton X-100, 10% glycerol, 10 mM Tris (pH 8), 150 mM sodium vanadate, 1 mM sodium fluoride, 5 mM EDTA, 1 mM DTT and cOmplete Protease Inhibitor Cocktail (Sigma)) and cell debris were removed by centrifugation. STAT3 was mixed with recombinant monobody at 10 µM for 8 h at 4 °C. Then, GST-proteins were added for an additional 16 h. Purification of GST was performed using GST SpinTrap columns (GE Healthcare Life Sciences) according to the manufacturer’s recommendations. Input and eluted samples were analyzed by western blot with an anti-STAT3 antibody (12640, CST). The membranes were then reprobed with anti-GST (RPN1236V, Sigma), anti-Flag tag antibody (F1804, Sigma) and anti-His tag antibody (12698, CST).

### Monobody expression and flow cytometry analysis

Ba/F3 cells were washed three times in PBS and 10^7^ cells were electroporated with the plasmid coding for monobody (15 µg) under specific conditions (74 Ω, 280 V, 1500 µF) and used after 4 h of incubation at 37 °C. A549-MS3-6 and A549-HA4-Y87A cells, which stably integrated the doxycycline inducible monobody construct, were plated (10^5^ cells) in six-well plates. The next day, cells were treated with control medium or Doxycycline (Sigma) at 1 µg ml^−1^ for 48 h. Cells were stimulated with control medium or with human IL-22 (500 ng ml^−1^) or human IL-6 (100 ng ml^−1^) for 15 min at 37 °C. After incubation with a viability marker (LIVE/DEAD Fixation Near-IR Dead Cell Stain Kit, Live), cells were fixed in paraformaldehyde 2% for 10 min at 37 °C and then permeabilized in 90% methanol for 30 min on ice. After two washes with PBS-EDTA 1% FCS, cells were stained with APC-labeled anti-STAT3-pY705 (557815, BD Biosciences) and AF488 labeled anti-cMyc tag (2279, CST) for 1 h at room temperature. Cells were analyzed on BD FACSVerse flow cytometer.

### Luciferase assay

For initial screening of the monobodies inhibitory activity, U3A-Luc cells stably expressing a STAT3 luciferase reporter system obtained from the D. Frank lab (Harvard) were used^62^. In all, 5 × 10^5^ cells were seeded in six-well plates and transfected upon reaching 60–80% confluency with a pEBTetD-eGFP-monobody vector using Polyfect (QIAGEN). In all, 1 µg ml^−1^ doxycycline was added to induce monobody expression for 48 h. Cells were next trypsinized and washed in FACS buffer (PBS, 0.5% BSA) and 8000 cells were directly sorted into 96-well plates in duplicate or triplicate using a SONY sorter SH800. Cells were then incubated overnight at 37 °C in 5% CO_2_ in the presence of doxycycline to ensure continuous monobody expression. The next morning, cells were stimulated using OSM 10 ng ml^−1^ for 6 h and luciferase activity was measured using the Bright Glo luciferase assay system (Promega) according to the manufacturer’s instructions. Untransfected cells treated with 6 µM Ruxolitinib for 7 h at 37 °C were used as positive control.

Additional Luciferase assays were done with 10^7^ BW5147 or Ba/F3 cells, which were electroporated with vector coding for the monobody (7,5 µg), pGL3-Pap1 luciferase reporter plasmid (7.5 µg) and pRL-TK plasmid (1 µg), as internal control of transfection, under specific conditions (74 Ω, 270 V, 1200 µF). Cells were then stimulated with control medium or with murine IL-9 (100 ng ml^−1^) or IL-24 (HEK293 supernatant 2%) or IFNλ3 (HEK293 supernatant 2%) for 4 h.

A549 with inducible monobody (HA4-Y87A or MS3-6) expression (5 × 10^3^ cells) were plated in a 96-well plate. After 24 h of treatment with Doxycycline (Sigma) at 1 µg ml^−1^, cells were transiently transfected using ViaFect (Promega), according to the manufacturer’s recommendations, with the pGL3-Pap1 luciferase reporter plasmid and pRL-TK plasmid. In all, 4 h after transfection, cells were stimulated with control medium or with human IL-22 (500 ng ml^−1^) or human IL-6 (100 ng ml^−1^) for 20 h at 37 °C. Luciferase activity was revealed with Dual-Glo Luciferase Assay System (Promega) according to the manufacturer’s recommendation.

### Reporting summary

Further information on research design is available in the Nature Research Reporting Summary linked to this article.

## Supplementary information

Supplementary Information

Reporting Summary

## Data Availability

The crystal structure of the MS3-6/STAT3-CF was deposited at Protein Data Bank under the accession code 6TLC. Proteomics data was deposited at ProteomeXchange with identifier PXD018374. Source data are provided with this paper. All other data are available from the corresponding author on reasonable request. Source data are provided with this paper.

## Source data

Source Data
