# Supplementary material for: Selective inhibition of STAT3 signaling using monobodies targeting the coiled-coil and N-terminal domains
Source: Nat Commun. 2020 Aug 17;11:4115. doi: 10.1038/s41467-020-17920-z (PMC7431413; doi:10.1038/s41467-020-17920-z)
Supplement: Supplementary file 1 — Supplementary Information [file 41467_2020_17920_MOESM1_ESM.pdf]

SUPPLEMENTARY INFORMATION

Selective inhibition of STAT3 signaling using monobodies targeting the coiled-coil and N-terminal domains

Grégory La Sala, et al.

Content

Supplementary Figures 1-6
Supplementary Tables 1-4

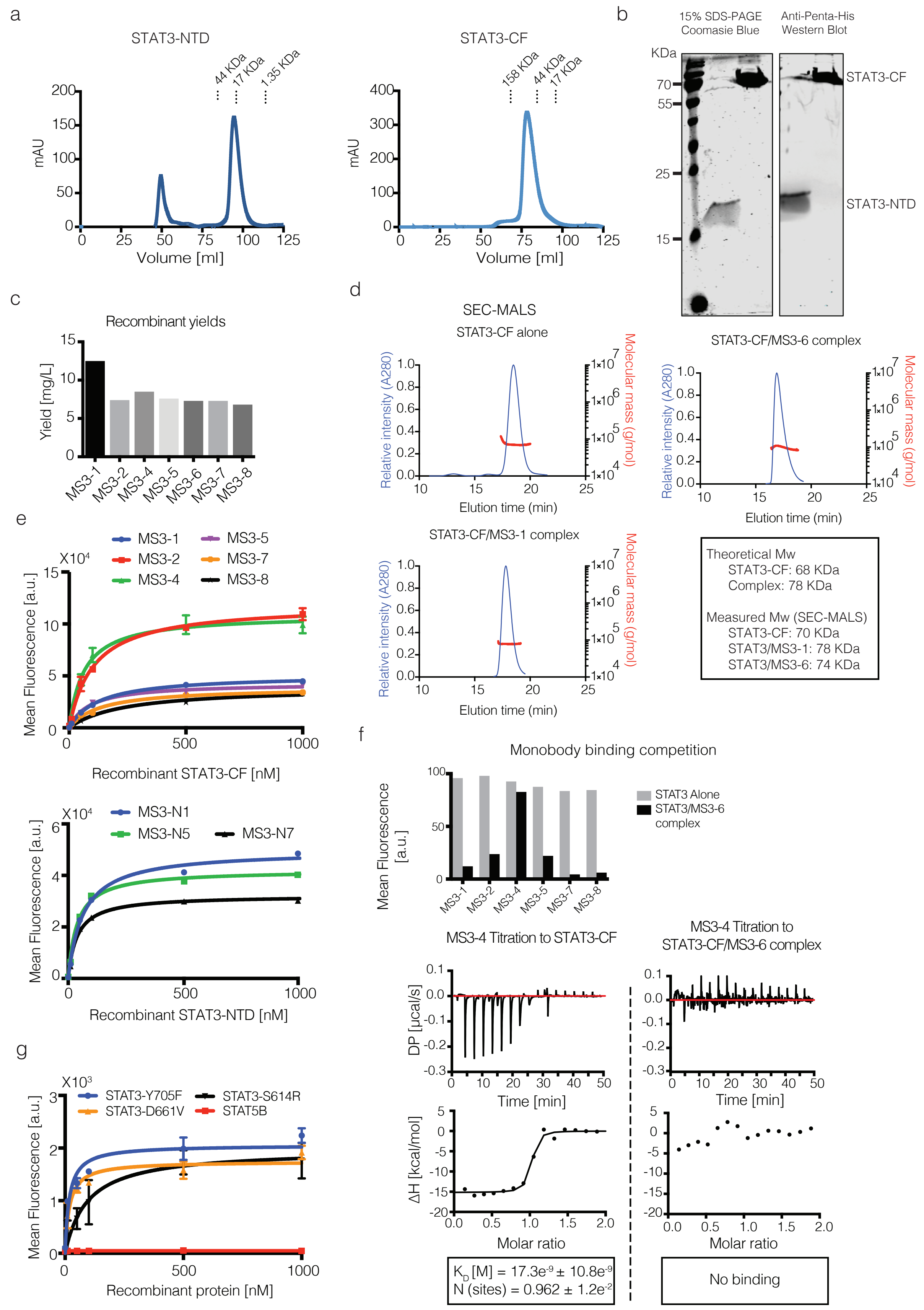

Supplementary Fig. 1: see next page for caption.

Supplementary Fig. 1: STAT3 target protein constructs and monobodies characterization.

(a) Size exclusion chromatography traces of the purified STAT3 proteins.
(b) Protein purity and identity was assessed by Coomassie staining and western blot analysis.
(c) Monobodies recombinant yields expressed in *E. coli* reported in mg of protein per liter of lysogeny broth (LB) expression medium.
(d) Gel filtration coupled with a multi-angle light-scattering analysis of STAT3-CF alone and in complex with monobodies.
(e) Yeast binding assay of all additional monobody clones selected against the STAT3-CF (upper panel) and STAT3-NTD (lower panel). Affinities are reported in Table 1. D ata are from one or two independent experiments (presented as mean ± SD).
(f) Pre-complex formation of STAT3-CF/MS3-6 prevents other monobodies to bind to STAT3 as measured by yeast binding assay (upper panel) with the exception of MS3-4. This can be explained as the affinities of MS3-6 and MS3-4 are in a comparable range. Hence, to determine whether MS3-6 and MS3-4 shared a similar epitope, a higher sensitivity technique (ITC) was used to demonstrate that MS3-4 does not bind to the pre-formed MS3-6 complex as well (f - lower panel). MS3-4 binding to STAT3-CF alone: ΔH (Kcal/mol) = -15.2 ± 0.5; ΔG (Kcal/mol) = -10.6.
(g) Binding of MS3-6 to STAT3 point mutants located in its SH2 domain identified in patients suffering from various hematological malignancies. Data presented as mean ± SD from two or three independent experiments.

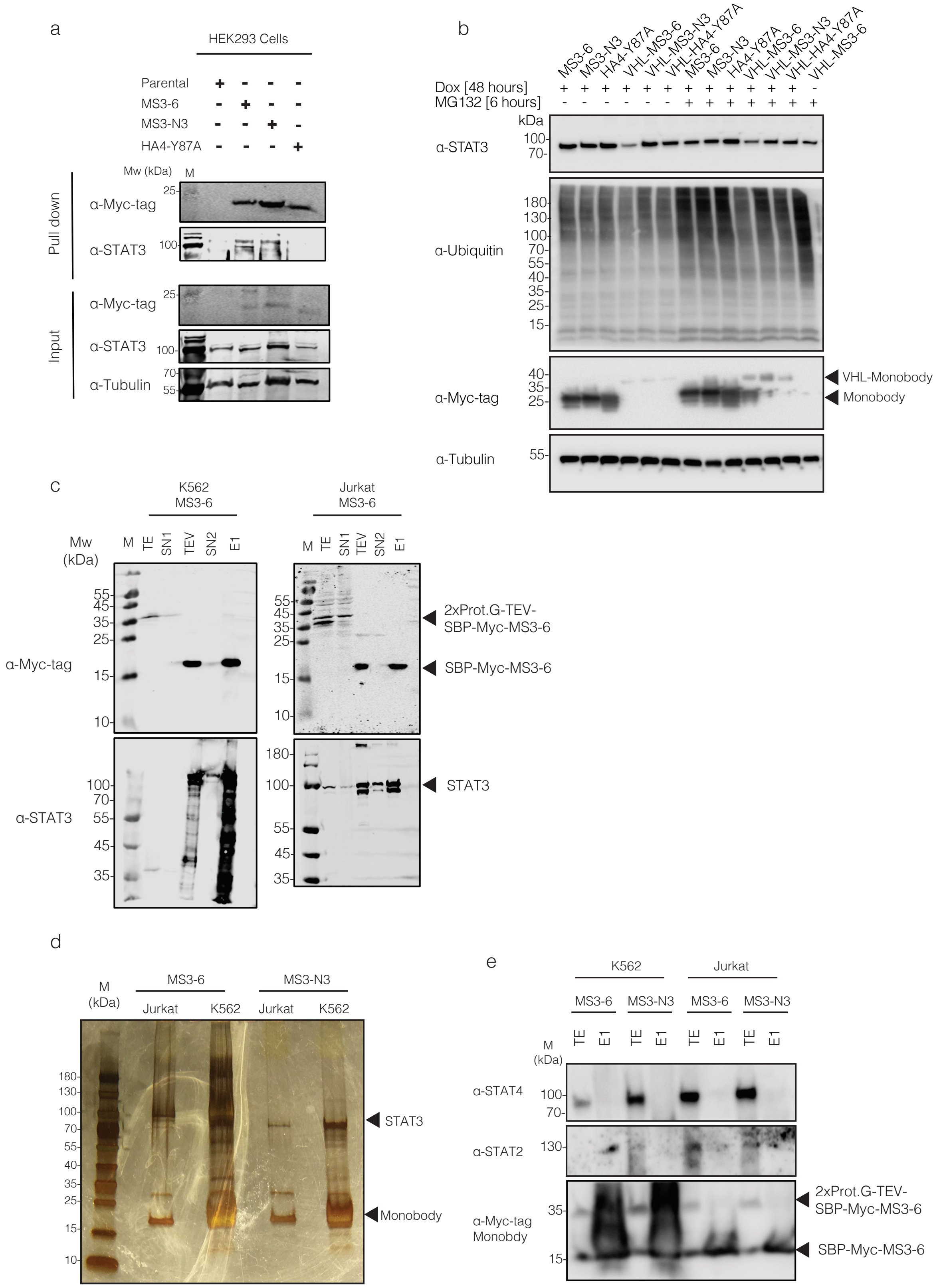

Supplementary Fig. 2: see next page for caption.

Supplementary Fig. 2: Monobodies MS3-6 and MS3-N3 binding to STAT3 in cellular contexts.

(a) Myc-tagged monobody pull down upon transient transfections in HEK293 cells was perform to assess STAT3 binding in the complex reducing cellular environment. STAT3 co-immunoprecipitation is detected with MS3-6 and MS3-N3, but not with HA4-Y87A control monobody.
(b) STAT3 protein levels in presence or absence of a proteasome inhibitor MG132 upon monobody or VHL-monobody expression. The VHL-MS3-6 mediated STAT3 degradation is impaired in presence of MG132, highlighting the implication of the proteasome in the STAT3 targeted degradation.
(c) Immunoblot analysis of Tandem-affinity purification (TAP) experiments from MS3-6 expression in Jurkat and K562 cells. Legends: M, Marker; TE, total extract; SN1 supernatant after protein G affinity capture; TEV, eluate after TEV cleavage; SN2, supernatant after streptavidin beads pull down; E1, eluate from streptavidin beads. Both monobody (bait) and STAT3 (target protein) were identified by immunoblotting using an anti-Myc tag and anti-STAT3 antibody detection.
(d) 10% of the eluate (E1) fraction were resolved by SDS-PAGE and visualized by silver staining to assess the overall sample purity for MS3-6 and MS3-N3 in Jurkat and K562 cells. Major bands corresponding to the monobodies and STAT3 were identified in all fractions.
(e) Total extracts (TE) and eluate fractions (E1) from Jurkat and K562 cells were probed for additional STAT family members. While both STAT2 and STAT4 are expressed at various levels in both cell lines, they could not be identified upon monobody pull down (E1 fractions).
All data presented result from single experiments.

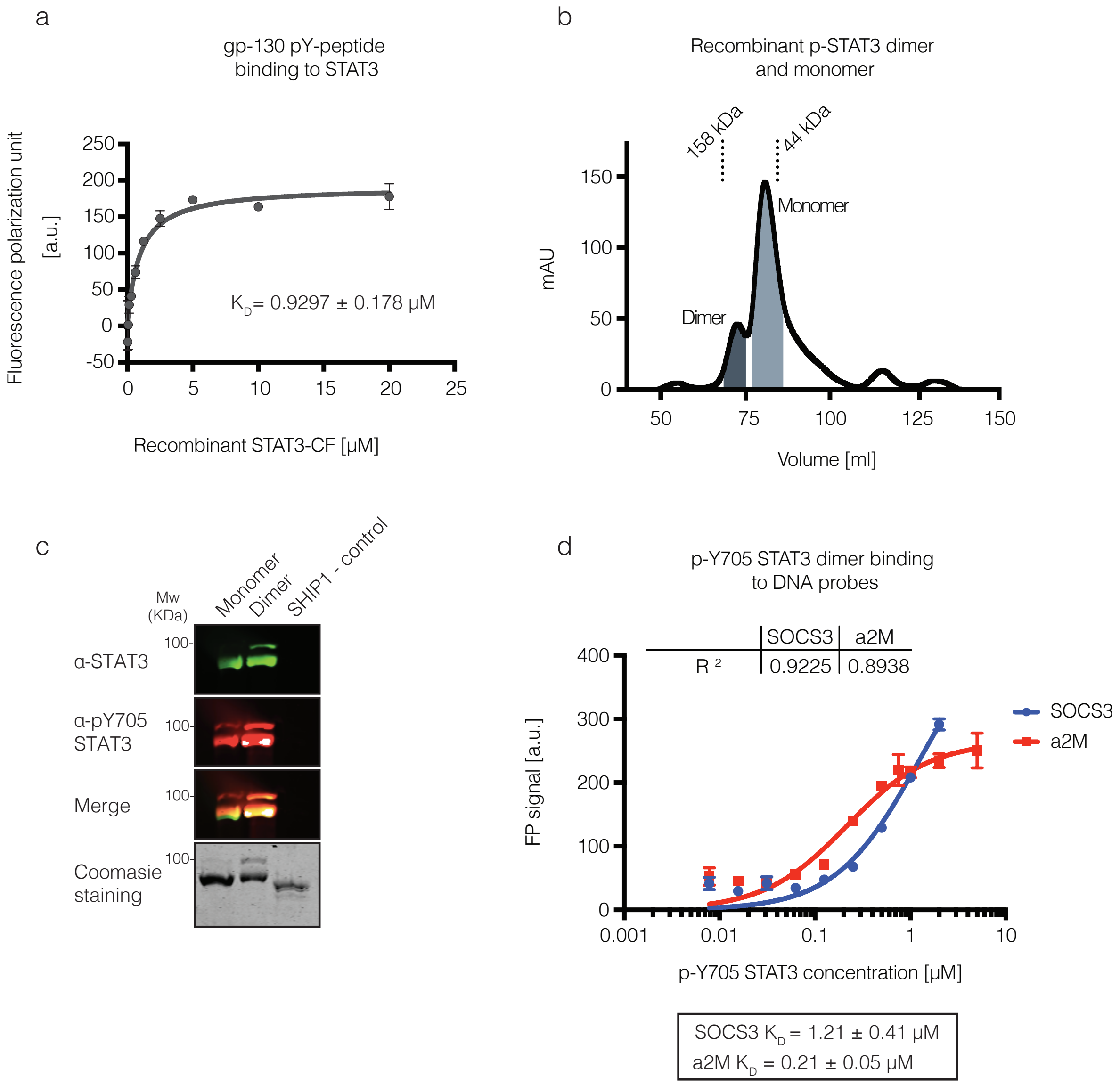

Supplementary Fig. 3: Recombinant p-Y705 STAT3 dimer and In vitro Fluorescent polarization assays.

(a) Fluorescence polarization experiments were performed to monitor the pY-gp130 peptide binding to STAT3 SH2 domain. Increasing concentrations of recombinant STAT3-CF was added to a 250nM peptide solution at 25°C. Data from two technical replicates.

(b) Size exclusion chromatography trace of a phospho-Y705 STAT3 expressed in E. coli. Monomeric and dimeric peaks are highlighted in light and dark blue respectively.

(c) STAT3 Y705 phosphorylation was observed by immunostaining. Recombinant SHIP1 was used as a negative control. Data from a single experiment.
(d) Fluorescence polarization experiments were performed to assess p-Y705 STAT3 dimer binding to two double stranded fluorescent DNA probes (full sequences in material and methods). Binding affinities are reported as K_D values. Data presented as mean ± SD from two independent experiments.

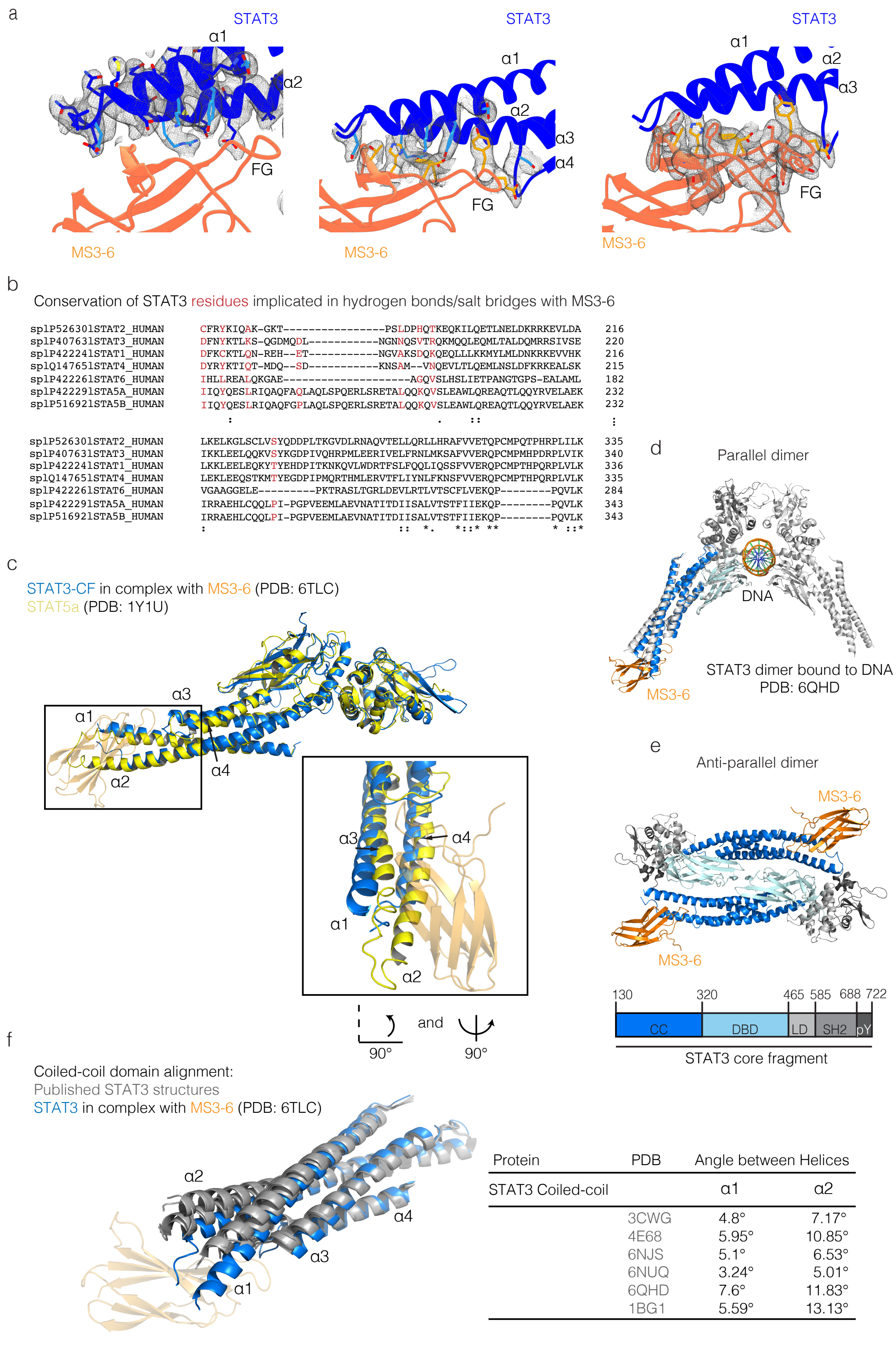

Supplementary Fig. 4: see next page for caption.

Supplementary Fig. 4: Structural alignment of the STAT3/MS3-6 complex with previously published STATs structures.

(a) Electron density map with solved model of STAT3 (blue) and MS3-6 (orange). Figure shows the 2mFo-DFc map at a 1 sigma contour level of the coiled-coil domain of STAT3 (left panel), of the binding interface (middle panel) and of MS3-6 (right panel).
(b) Sequence alignment of all STAT family members. Residues implicated in MS3-6 binding are highlighted in red.
(c) Alignment of STAT3 (blue)/MS3-6 (orange) complex (PDB: 6TLC) with a STAT5a (yellow) structure (PDB: 1Y1U). The magnification of the monobody binding interface highlights the torsion of the STAT3 coiled-coil domain (blue), as compared to the STAT5a coiled-coil domain (yellow). The longer helix α2 of STAT5a clashes with monobody binding (shown in transparency).
(d) Structural alignment of the STAT3/MS3-6 complex with a STAT3 dimer bound to DNA (PDB: 6QHD) highlight the compatibility of the monobody binding with an efficient p-Y705 STAT3 parallel dimer formation. Individual domains are color coded as indicated.
(e) Formation of an anti-parallel STAT3 dimer, as observed in the crystal symmetry unit, is compatible with monobody binding. Individual domains are color coded as indicated.
(f) MS3-6 binding to STAT3 (blue) leads to the torsion of the helices α1 and α2 of the coiled-coil domain as evidenced by the structural alignment with previously published STAT3 structures (in grey). Orientation vectors (Pymol) for each helices were used to measure the angle formed between the respective structures and are reported as a table.

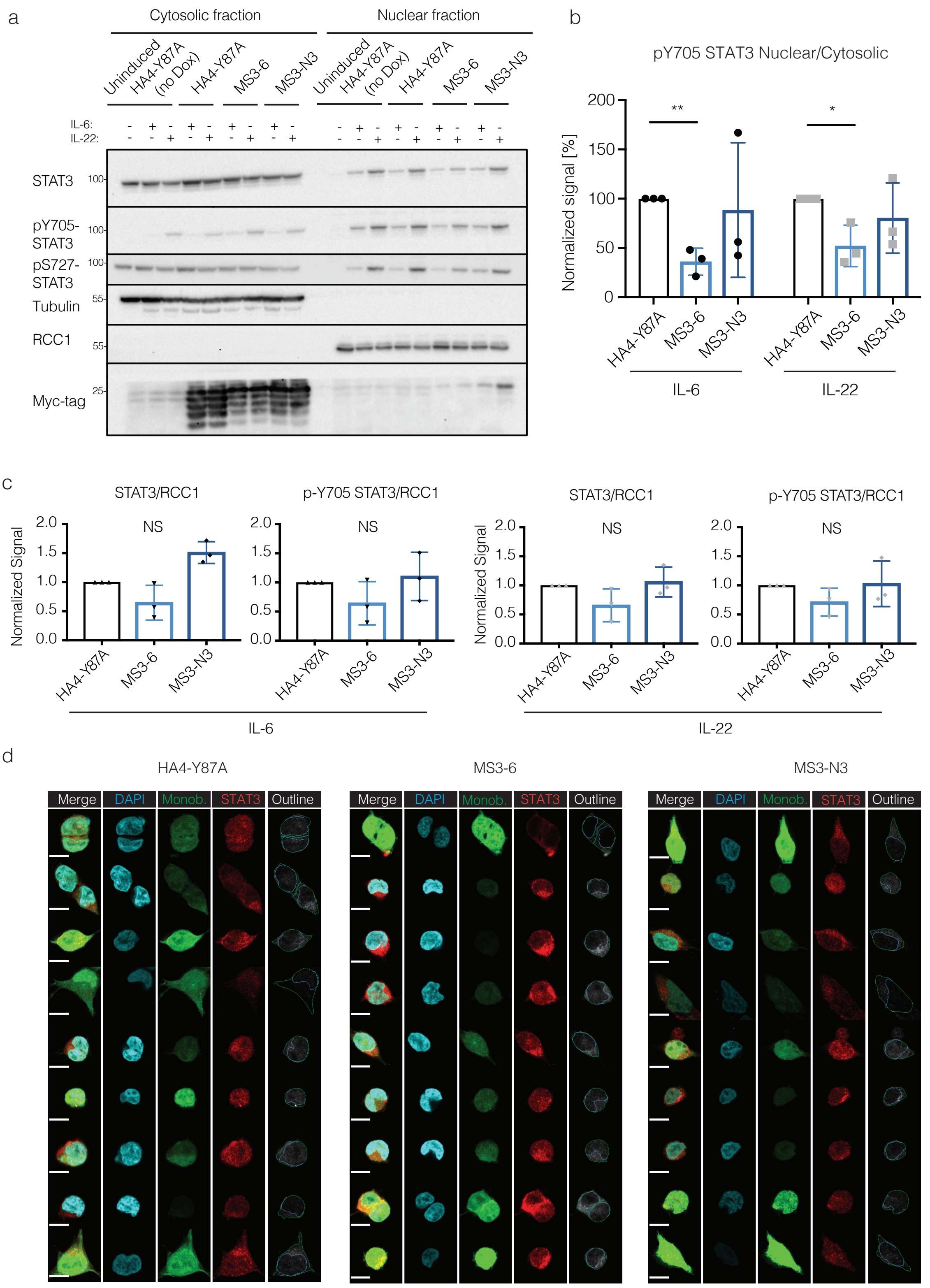

Supplementary Fig. 5: see next page for caption.

Supplementary Fig. 5: MS3-6 decreases the nuclear/cytosolic STAT3 ratio upon cytokines stimulation.

(a) Representative immunoblot analysis of STAT3 nuclear translocation upon cytokine stimulation. MS3-6 reduces STAT3 nuclear levels as compared to the HA4-Y87A or no monobody (uninduced) control conditions. Experiment performed in three independent replicates.
(b) Quantification of three independent immunoblot analysis of cellular fractionation experiments. A549 cells were treated with doxycycline to induce monobody expression for 48h, followed by IL-6 or IL-22 stimulation. Significance according to a two-tailed unpaired *t*-test analysis: *P = 0.0168, **P = 0.0013.
(c) Quantification of three independent immunoblot analysis of cellular fractionation experiments according to the protein levels loaded (RCC1 signal).
(d) Additional representative images from confocal microscopy experiments performed in two independent experiments using HEK293 cells transiently transfected with a doxycycline inducible eGFP-monobody fusion and stimulated with IL-6 for 20 minutes at 37°C. Scale bar represents 10μm. The outline panel illustrates the threshold defined (CellProfiler) to determine the nucleus (blue line) and cytosolic compartments (green line).

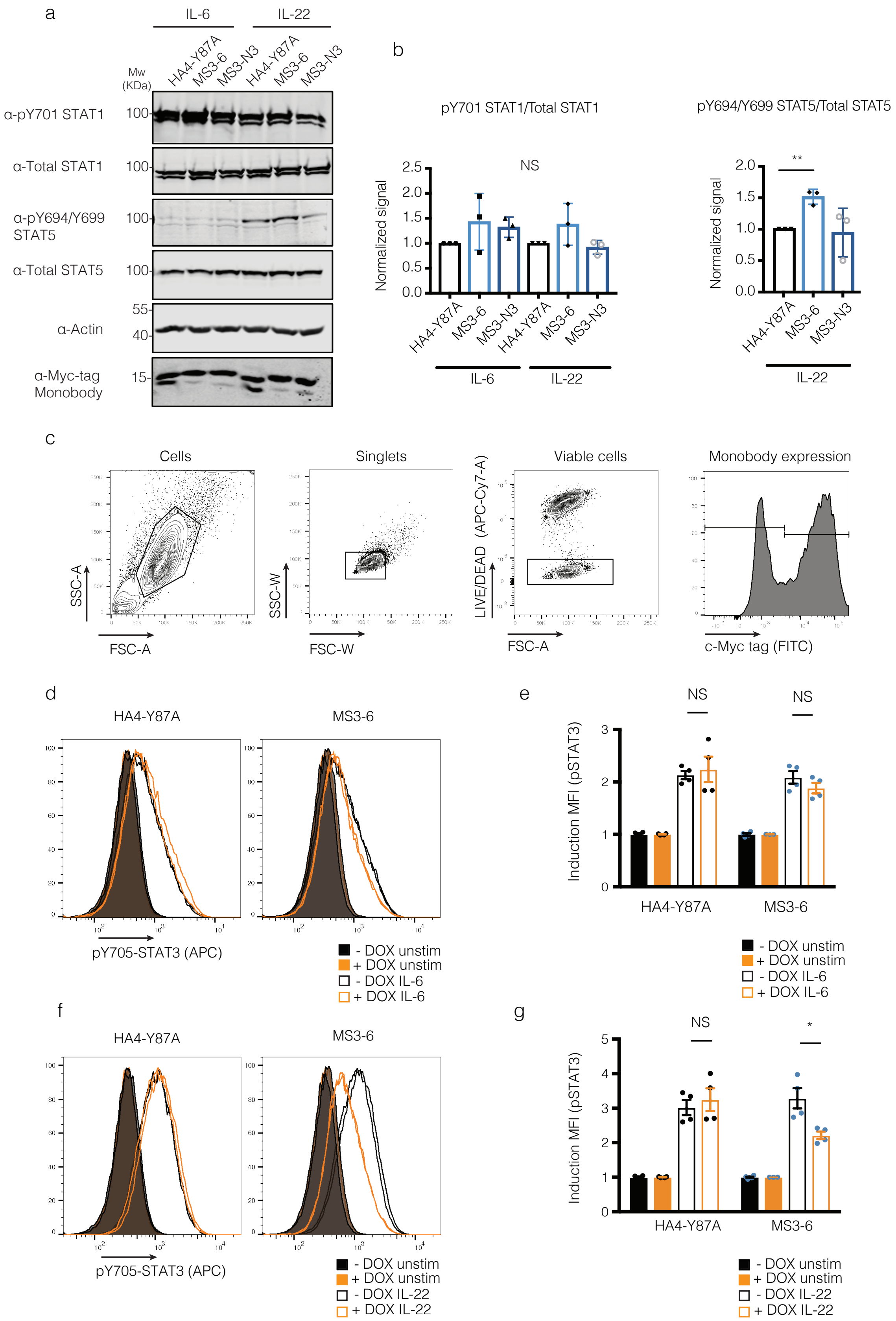

Supplementary Fig. 6: see next page for caption.

Supplementary Fig. 6: MS3-6 reduces STAT3 p-Y705 levels upon IL-22 stimulation.

(a) Representative immunoblot analysis of STAT1 and STAT5 phosphorylation levels following stimulation with IL-6 and IL-22 in presence of monobodies. Data representative of three independent experiments. Actin, total STAT1/p-Y701 STAT1 and total STAT5/pY-694 -699 STAT5 blots were processed in parallel. **(b)** Quantification of STAT1 and STAT5 phosphorylation levels upon IL-6 or IL-22 stimulation in presence of monobodies. Quantifications were performed out of three independent experiments. Significance according to a two-tailed unpaired t-test analysis: **P = 0.0016.
(c) Flow cytometry gating strategy applied for all FACS data analysis. Viable cells were analyzed for monobody expression following doublets exclusion.
(d) and **(f)** Flow cytometry on A549 cells with inducible monobody (HA4-Y87A or MS3-6) expression. Cells were treated with control or doxycycline (1 μg/ml) containing medium to induce monobody expression. Cells were stimulated with IL-6 (100 ng/ml) **(e)** or IL-22 (500 ng/ml) **(g)** and intracellular FACS staining was performed.
(e) and **(g)** phospho-Y705 STAT3 levels were assessed, quantified and plotted as mean fluorescence intensities. Data are presented as mean ± SEM of two independent experiments performed in duplicates. Significance according to two-tailed unpaired *t*-test analysis: *P = 0.014.

Supplementary Tables 1-4.

Complete list of primers, including names and sequences

Supplementary Table 1: Primers used to clone the core fragment (CF) and NTD of STAT3.

**hSTAT3-CF [aa 129-722]**	Forward	CATGGGATCCGGCCAGGCCAACCACC
	Reverse	CATGCTCGAGTCAAATGGTATTGCTGCAGGTCGTTG
**hSTAT3-NTD [aa 3-138]**	Forward	CATGGGATCCCAATGGAATCAGCTACAGCAGCTTG
	Reverse	CATGCTCGAGTCACGTCACCACGGCTGC
**hSTAT5B [aa 129 - 714]**	Forward	CATGGGATCC CCA GCT GGA AGC CTT GCT G
	Reverse	CTGCTCGAG TCA TGC GTT CAC AAA CTC AGG GAC

Supplementary Table 2: STAT3 oncogenic point mutants site directs mutagenesis primers.

**STAT3-S614R**	Forward	GATTCAGTGAAAGCAGGAAAGAAGGAGGCGTC
	Reverse	GACGCCTCCTTCTTTCCTGCTTTCACTGAATC
**STAT3-D661V**	Forward	GCTATAAGATCATGGTTGCTACCAATATCCTG
	Reverse	CAGGATATTGGTAGCAACCATGATCTTATAGC
**STAT3 Y705F**	Forward	GTAGCGCTGCCCCATTCCTGAAGACCAAGTTTATC
	Reverse	GATAAACTTGGTCTTCAGGAATGGGGCAGCGCTAC

Supplementary Table 3: Universal monobody gateway cloning primers.

**For**	GGGGACAAGTTTGTACAAAAAAGCAGGCTCCATGGTTTCTTCTGTTCCGACCAAAC
**Rev**	GGGGACCACTTTGTACAAGAAAGCTGGGTCCTAGGTACGGTAGTTAATCGAG

Supplementary Table 4: List of all vectors used.

Vector name	Description
**pHBT**	Modified pET vector, N-term 6xHis-Avi-tag-TEV-BamHI-XhoI, used for bacterial expression of recombinant proteins inserted using BamHI/XhoI
**pEM24**	Modified pCW2239 obtained from E. Meylan, EPFL. Doxycycline inducible, N term 6xMyc-tagged, gateway vector, used for dox induced monobody expression in cells.
**pCMV-R8_74**	Lentiviral expression system encoding gag and pol proteins
**pMD2_G**	Lentiviral expression system encoding VSV-G envelope
**pCMV-VSV-G**	Retroviral expression system encoding VSV-G envelope. Obtained from the Superti-Furga lab
**pRV-NTAP**	2xProteinG-TEV-cMyc tag. Gateway vector. Used for tandem affinity purification pull downs.
**pEBtetD**	Doxycycline inducible, N-term eGFP-tag. Used for luciferase assays and immunofluorescence.
**pCS2**	N term 6x-Myc tag. Gateway vector. Used for transient transfections and luciferase assays.
