# Supplementary material for: Selective inhibition of STAT3 signaling using monobodies targeting the coiled-coil and N-terminal domains
Source: Nat Commun. 2020 Aug 17;11:4115. doi: 10.1038/s41467-020-17920-z (PMC7431413; doi:10.1038/s41467-020-17920-z)
Supplement: Supplementary file 2 — Reporting Summary [file 41467_2020_17920_MOESM2_ESM.pdf]

nature research

Corresponding author(s): Oliver Hantschel

Last updated by author(s): 10.07.2020

Reporting Summary

Nature Research wishes to improve the reproducibility of the work that we publish. This form provides structure for consistency and transparency in reporting. For further information on Nature Research policies, see Authors & Referees and the Editorial Policy Checklist .

Statistics

For all statistical analyses, confirm that the following items are present in the figure legend, table legend, main text, or Methods section.

n/a	Confirmed	
	x	The exact sample size (*n*) for each experimental group/condition, given as a discrete number and unit of measurement
	x	A statement on whether measurements were taken from distinct samples or whether the same sample was measured repeatedly
	x	The statistical test(s) used AND whether they are one- or two-sided<br>*Only common tests should be described solely by name; describe more complex techniques in the Methods section.*
x		A description of all covariates tested
x		A description of any assumptions or corrections, such as tests of normality and adjustment for multiple comparisons
	x	A full description of the statistical parameters including central tendency (e.g. means) or other basic estimates (e.g. regression coefficient) AND variation (e.g. standard deviation) or associated estimates of uncertainty (e.g. confidence intervals)
	x	For null hypothesis testing, the test statistic (e.g. *F*, *t*, *r*) with confidence intervals, effect sizes, degrees of freedom and *P* value noted<br>*Give P values as exact values whenever suitable.*
x		For Bayesian analysis, information on the choice of priors and Markov chain Monte Carlo settings
x		For hierarchical and complex designs, identification of the appropriate level for tests and full reporting of outcomes
x		Estimates of effect sizes (e.g. Cohen's *d*, Pearson's *r*), indicating how they were calculated

Our web collection on statistics for biologists contains articles on many of the points above.

Software and code

Policy information about availability of computer code

Data collection	Crystallographic diffraction data were processed and scaled with the XDS package (Kabsch, 1993).
Data analysis	Proteomics data were processed using Proteome Discoverer V2.2. Data was inspected using Scaffold4.10.0.<br>Confocal microscopy images were analysed using Cellprofiler 3.1.9.<br>Flow cytometry data was anaylsed using FlowJo v10.7.<br>Crystallography software: Molecular replacement, manual model building, B-factor refinement, solvent addition, energy-minimization: Phaser and Coot (Phenix version 1.17.1); Refinement of structures using REFMAC v 5.8; Molecular graphics: PyMOL 2.0.4 (DeLano Scientific).

For manuscripts utilizing custom algorithms or software that are central to the research but not yet described in published literature, software must be made available to editors/reviewers. We strongly encourage code deposition in a community repository (e.g. GitHub). See the Nature Research guidelines for submitting code & software for further information.

Data

Policy information about availability of data

All manuscripts must include a data availability statement. This statement should provide the following information, where applicable:

- Accession codes, unique identifiers, or web links for publicly available datasets
- A list of figures that have associated raw data
- A description of any restrictions on data availability

The crystal structure of the MS3-6/STAT3-CF was deposited at Protein Data Bank (entry 6TLC). Proteomics data was deposited at ProteomeXchange with identifier PXD018374. Source data are provided as a Source Data file.

Field-specific reporting

Please select the one below that is the best fit for your research. If you are not sure, read the appropriate sections before making your selection.

- [x] Life sciences
- [ ] Behavioural & social sciences
- [ ] Ecological, evolutionary & environmental sciences

For a reference copy of the document with all sections, see nature.com/documents/nr-reporting-summary-flat.pdf

Life sciences study design

All studies must disclose on these points even when the disclosure is negative.

Sample size	No sample size calculation was applied in this study to predetermine sample sizes for experiments using cell lines. A sample size of three was used as a starting point to evaulate the spread of the data (Casadevall A, Fang FC; Reproducible Science, Infect Immun, 2010 Dec; 78 (12):4972-4975). Experiments were repeated more often if necessary to provide results with statistical significance.
Data exclusions	Immunofluorescence microscopy: cells whose detection failed using the defined CellProfiler workflow as detailed in the material and methods were excluded
Replication	All experiments were replicated as stated in the figure legends
Randomization	Immunofluorescence microscopy: cells were randomly chosen for imaging and subsequent quantification. No randomization is applicable for flow cytometry as cells positive for Monobody expression are analysed. For other experiments randomization is not relevant as cells in bulk were used.
Blinding	No blinding, as the same investigator performed most experiments and analyzed the data

Reporting for specific materials, systems and methods

We require information from authors about some types of materials, experimental systems and methods used in many studies. Here, indicate whether each material, system or method listed is relevant to your study. If you are not sure if a list item applies to your research, read the appropriate section before selecting a response.

Materials & experimental systems

n/a	Involved in the study	
☐	☒	Antibodies
☐	☒	Eukaryotic cell lines
☒	☐	Palaeontology
☒	☐	Animals and other organisms
☒	☐	Human research participants
☒	☐	Clinical data

Methods

n/a	Involved in the study	
☒	☐	ChIP-seq
☐	☒	Flow cytometry
☒	☐	MRI-based neuroimaging

Antibodies

Antibodies used

STAT1: Rabbit monoclonal anti-STAT1 Cell Signaling Technology Cat# 9172, RRID:AB_2198300
pY701 STAT1: Rabbit monoclonal pY701 STAT1 Cell Signaling Technology Cat# 9167, RRID:AB_561284
STAT2: Rabbit polyclonal STAT2, Cell Signaling Technology Cat# 4597, RRID:AB_2198305
STAT3: Mouse monoclonal STAT3 Cell Signaling Technology Cat# 9139, RRID:AB_331757
pY705 STAT3: Rabbit monoclonal pY705 STAT3 Cell Signaling Technology Cat# 9145, RRID:AB_2491009
pS727 STAT3: Rabbit polyclonal pS727 STAT3 Cell Signaling Technology Cat# 9134, RRID:AB_331589
STAT4: Rabbit monoclonal STAT4, Cell Signaling Technology Cat# 2653, RRID:AB_2255156
pY694/pY699 STAT5: Rabbit monoclonal pY694 STAT5 Cell Signaling Technology Cat# 9359, RRID:AB_823649
STAT5B: Mouse monoclonal STAT5b Santa Cruz Biotechnology Cat# sc-1656, RRID:AB_2197067
ubiquitin antibody: Mouse Anti-Ubiquitinylated proteins Monoclonal antibody, Unconjugated, Clone fk2. Millipore Cat# 04-263, RRID:AB_612093
anti-Flag: Mouse monoclonal anti-Flag, Sigma-Aldrich Cat# F1804, RRID:AB_262044
penta-His: Mouse monoclonal anti-Penta-his, Qiagen, Cat# 34660; RRID: AB_2619735
anti-alpha Tubulin antibody: Mouse monoclonal anti-Tubulin, Sigma, Cat# T9026; RRID: AB_477593
anti-Myc tag: Mouse monoclonal anti-Myc-tag Myc.A7 DyLight800, Thermo Scientific, Cat# MA1-21316-D800; RRID: AB_2536996
anti mouse IRDye800: Goat polyclonal anti-mouse IgG IRDye 800CW, LiCor, P/N 926-32210; RRID: AB_621842
anti mouse IRDye680: Goat anti-Mouse igG IRDye 680RD secondary antibody, LiCor, P/N 926-68070: RRID AB_10956588
anti rabbit IRDye680: Goat polyclonal anti-Rabbit IgG IRDye680, LI-COR Biosciences Cat# 926-68071, RRID:AB_10956166

	anti rabbit HRP-coupled: Goat Anti-rabbit IgG, HRP-linked Antibody, Cell Signaling Technology Cat# 7074, RRID:AB_2099233<br>anti mouse HRP-coupled: Horse Anti-mouse IgG, HRP-linked Antibody, Cell Signaling Technology Cat# 7076, RRID:AB_330924
Validation	All used antibodies were validated commercially. Certificates of analysis for the approved applications by the manufacturer are available on the company websites.

Eukaryotic cell lines

Policy information about cell lines

Cell line source(s)	HEK293, ATCC, Cat# CRL-1573; RRID: CVCL_0045<br>U3A-Luc, Gifted from David A. Frank's lab, Harvard. Originally from ECACC 12021503, CVCL_9469, 2fTGH-U3A (RRID:CVCL_9469)<br>A549, Gifted from Etienne Meylan's Lab, EPFL, Originally purchased at ATCC Cat# CCL-185, RRID:CVCL_0023<br>K562, DSMZ Cat# ACC-10, RRID:CVCL_0004<br>Jurkat, DSMZ Cat# ACC-282, RRID:CVCL_0065<br>Ba/F3, DSMZ Cat# ACC 300, RRID:CVCL_0161<br>BW5147, TKG Cat# TKG 0729 RRID:CVCL_3896
Authentication	cell lines from ATCC , ECACC and DSMZ were authenticated by the vendors (STR profiling and DNA barcoding).
Mycoplasma contamination	cell lines were regularly tested and were mycoplama negative
Commonly misidentified lines<br>(See ICLAC register)	no commonly misidentified cell lines were used in the study.

Flow Cytometry

Plots

Confirm that:

- [x] The axis labels state the marker and fluorochrome used (e.g. CD4-FITC).
- [x] The axis scales are clearly visible. Include numbers along axes only for bottom left plot of group (a 'group' is an analysis of identical markers).
- [x] All plots are contour plots with outliers or pseudocolor plots.
- [x] A numerical value for number of cells or percentage (with statistics) is provided.

Methodology

Sample preparation	Ba/F3 and A549 cells were stimulated as described in the methods section, cells were fixed in in Paraformaldehyde 2% for 10 min at 37°C and then permeabilized in 90% methanol for 30 min on ice.
Instrument	BD FACSVerse
Software	FlowJo V10
Cell population abundance	Populations transiently expressing monobodies upon vector electroporation or transfection were monitored using an intracellular Myc-Tag AF488 detection. Abundance of cells expressing the monobodies varied across experiments due to varying transfection efficiencies.
Gating strategy	Viable cells were analyzed based on a viability marker (LIVE/DEAD Fixation Near-IR Dead Cell Stain Kit, Live). Monobody expression was monitored based on a Myc-tag AF488 detection.

- [x] Tick this box to confirm that a figure exemplifying the gating strategy is provided in the Supplementary Information.
